# B‐GATA factors are required for nitrogen‐responsive growth in *Physcomitrium patens* and *Arabidopsis thaliana*

**DOI:** 10.1111/nph.70887

**Published:** 2026-01-09

**Authors:** Dario Zappone, Peter Michael Schröder, Ivan Petřík, Xiao Dong, Rudi Schäufele, Korbinian Schneeberger, Ondřej Novák, Claus Schwechheimer

**Affiliations:** ^1^ Plant Systems Biology, School of Life Sciences Technical University of Munich 85354 Freising Germany; ^2^ Laboratory of Growth Regulators, Faculty of Science Palacký University and Institute of Experimental Botany, Czech Academy of Sciences Šlechtitelů 27 77900 Olomouc Czech Republic; ^3^ Department of Chromosome Biology Max Planck Institute for Plant Breeding Research 50829 Cologne Germany; ^4^ Professorship of Crop Physiology, School of Life Sciences Technical University of Munich 85354 Freising Germany; ^5^ Faculty of Biology LMU Munich 82152 Planegg‐Martinsried Germany; ^6^ Cluster of Excellence on Plant Sciences Heinrich‐Heine University 40225 Düsseldorf Germany

**Keywords:** *Arabidopsis thaliana*, cytokinin, GATA factor, moss, nitrogen, *Physcomitrium patens*, vascular plant

## Abstract

- We hypothesized that B‐GATA family transcription factors have important roles in growth regulation in moss.
- We analyzed B‐GATA family transcription factor mutants from *Physcomitrium patens* and *Arabidopsis thaliana* to assess growth, gene expression, and cytokinin‐related processes under varying nitrogen conditions.
- We found that nitrogen‐dependent growth and transcriptional regulation are strongly impaired in mutants from *Physcomitrium* and *Arabidopsis*. We detected altered cytokinin homeostasis or signaling in the mutants, linking hormonal imbalance to growth and transcription defects.
- We demonstrated a conserved, critical role of B‐GATAs in plant nitrogen‐responsive growth. Results suggest that B‐GATAs influence nitrogen‐regulated transcription downstream from cytokinin, supporting an ancient, evolutionarily conserved mechanism connecting nutrient signaling to growth. We provided experimental evidence for the long‐speculated but as‐yet not demonstrated role of GATA transcription factors in nitrogen‐dependent growth in land plants.

## Introduction

GATA transcription factors are evolutionarily conserved zinc finger proteins that preferentially bind to the DNA sequence G‐A‐T‐W (Lowry & Atchley, 2000). While animal and fungal genomes typically encode only a few GATA factors, for example six in humans and four in the yeast *Saccharomyces cerevisiae*, the *GATA* gene family is notably expanded in land plants, with close to 30 members in *Arabidopsis thaliana* (Schwechheimer *et al*., 2022). In vascular plants, GATA factors can be classified into four subgroups, classes A, B, C, and D, based on differences in their zinc finger DNA‐binding domains and the presence of additional protein domains at their N‐ or C‐termini (Schwechheimer *et al*., 2022).

In the angiosperm *Arabidopsis*, class B GATA factors are further subdivided into HAN‐ and LLM‐domain B‐GATAs (R. Richter *et al*., 2010; Behringer & Schwechheimer, 2015; Schwechheimer *et al*., 2022). The LLM‐domain is defined by a conserved C‐terminal leucine–leucine–methionine motif of unknown function that is essential for full GATA activity (Behringer *et al*., 2014; Schwechheimer *et al*., 2022). Among the six *Arabidopsis* LLM‐domain B‐GATAs, *GNC* (*GATA*, *NITRATE‐INDUCIBLE*, *CARBON‐METABOLISM‐RELATED*) and its paralog *GNL* (*GNC‐LIKE*) are the best studied. Their expression is regulated by the phytohormones gibberellin, cytokinin, and auxin, as well as by light (Naito *et al*., 2007; R. Richter *et al*., 2010, 2013). *GNC* and *GNL*, together with *GATA15*, *GATA16*, *GATA17*, and *GATA17L*, contribute to key physiological processes including chloroplast development, Chl and starch biosynthesis, as well as stomatal patterning (R. Richter *et al*., 2010; Chiang *et al*., 2012; Klermund *et al*., 2016; Ranftl *et al*., 2016; Bastakis *et al*., 2018; Zubo *et al*., 2018). HAN‐domain B‐GATAs contain a short, conserved domain of unknown function, initially characterized in the *han* (*hanaba taranu*) mutant (Zhao *et al*., 2004). Mutations in *HAN* cause floral abnormalities, while double and triple mutants of *HAN*, *HAN‐LIKE1*, and *HAN‐LIKE2* exhibit severe embryonic defects (Zhao *et al*., 2004; Nawy *et al*., 2010; Whipple *et al*., 2010).

Interestingly, the HAN‐ and LLM‐based subdivision of angiosperm B‐GATAs does not apply to bryophytes. The liverwort *Marchantia polymorpha* and the moss *Physcomitrium patens* encode one and four B‐GATA genes, respectively, each harboring LLM‐ and HAN‐domains (Schwechheimer *et al*., 2022; Schroder *et al*., 2023). In *Marchantia*, the single B‐GATA, *MpB‐GATA*, promotes greening and protects against high‐light stress (Schroder *et al*., 2023). However, the biological roles of the four B‐GATAs from *Physcomitrium* remain largely uncharacterized (Schroder *et al*., 2023).

Nitrogen (N) is an essential macronutrient for plant growth, typically acquired as nitrate (NO_3_^−^) or ammonium (NH_4_^+^). The molecular and physiological responses to NO_3_^−^ have been extensively studied: its uptake is mediated by nitrate transporters (NRTs), primarily from the low‐affinity NRT1, in *Arabidopsis* NRT1.1/CHL1 and NRT1.2, and high‐affinity NRT2 families, in *Arabidopsis* NRT2.1 and NRT2.2 (Tsay *et al*., 1993; Filleur *et al*., 2001; Wang *et al*., 2018; Lamig *et al*., 2022). NRT1.1 functions as a dual‐affinity transporter for high‐ and low‐affinity uptake depending on its phosphorylation status (Liu & Tsay, 2003). NH_4_^+^ uptake is facilitated by ammonium transporters (AMTs) (Ninnemann *et al*., 1994; Williamson *et al*., 2024).

Within the plant, NO_3_^−^ is reduced to NO_2_^−^ by cytosolic nitrate reductase (NR), NIA1 and NIA2 in *Arabidopsis*, and further to NH_4_^+^ by chloroplastic nitrite reductase (NiR). The resulting NH_4_^+^ is assimilated into amino acids, Chl, and citric acid cycle intermediates via the GS/GOGAT cycle, involving glutamine synthetase (GS), glutamate synthase (GOGAT), and glutamate dehydrogenase (GDH) (x. Liu *et al*., 2022). N uptake triggers signaling events, including changes in cytoplasmic calcium levels, activation of calcium‐dependent protein kinases, and transcriptional responses, for example coordinated by NIN‐LIKE PROTEIN 7 (NLP7), which has been proposed to act as a nitrate sensor (Castaings *et al*., 2009; Marchive *et al*., 2013; Ristova *et al*., 2016; Ruffel *et al*., 2016; K. H. Liu *et al*., 2017, 2022; Varala *et al*., 2018; Brooks *et al*., 2019). Cytokinin contributes to N‐responsive growth by modulating these signaling pathways (Takei *et al*., 2002; Sakakibara *et al*., 2006; Landrein *et al*., 2018).

In fungi, GATA factors play pivotal roles in N regulation. In *Aspergillus nidulans*, the GATA factor AreA controls the expression of genes involved in N uptake and catabolism (Arst & Cove, 1973; Chudzicka‐Ormaniec *et al*., 2019). Under N‐rich conditions, AreA mediates N metabolite repression, suppressing enzymes and transporters for poor N sources (e.g. proline, allantoin, GABA) when superior N sources (e.g. glutamine, asparagine) are available (Caddick, 1994; Hofman‐Bang, 1999; Georis *et al*., 2009). AreA also regulates genes for N catabolism and NH_4_^+^ transport (Caddick, 1994; Monahan *et al*., 2006). AreB, another GATA factor in *A. nidulans*, represses formidase (fmdS) and genes involved in arginine catabolism under NH_4_^+^‐rich conditions (Chudzicka‐Ormaniec *et al*., 2019). In *Saccharomyces cerevisiae*, four GATA factors, Gln3, Nil1, Nil2, and Dal80, modulate N metabolite repression, with Gln3 and Nil1 acting as activators and Nil2 and Dal80 as repressors (Hofman‐Bang, 1999; Cooper, 2002; Magasanik, 2005; Georis *et al*., 2009).

Inspired by fungal studies, researchers have long sought links between plant GATAs and N metabolism. The *Arabidopsis* B‐class transcription factor GNC was initially identified as nitrate‐inducible, giving rise to its name *GATA NITRATE‐INDUCIBLE CARBON‐METABOLISM‐RELATED* (Scheible *et al*., 2004; Bi *et al*., 2005). *GNC* transcript levels increase 2 h after transferring seedlings from glutamine‐containing to NO_3_^−^‐containing media (Bi *et al*., 2005). Furthermore, *GATA17L* has been implicated in N response as a result of a comprehensive transcriptional network study (Ristova *et al*., 2016). However, a direct role of B‐GATAs in N‐regulated growth in *Arabidopsis* has not been conclusively demonstrated.

In *Physcomitrium*, growth begins with germination of spores into a filamentous protonema, composed of chloronemal and caulonemal cells, which later give rise to leafy gametophores. This stage is essential for nutrient uptake and surface colonization. While the influence of N on *Physcomitrium* growth is not well understood, prior studies have examined NO_3_^−^ transporter expression and genome‐wide transcriptional changes upon NH_4_^+^ supplementation, reporting repression of genes involved in NO_3_^−^ and NH_4_^+^ assimilation and NO_3_^−^ metabolism (Tsujimoto *et al*., 2007; Perroud *et al*., 2018).

Based on the important role of B‐GATA factors in plant growth and development in the angiosperm *Arabidopsis* and the bryophyte *Marchantia*, we speculated that *B‐GATA* genes may also have an important function in mosses. To examine this hypothesis, we investigated the *Physcomitrium B‐GATA* genes (*PpB‐GATA1*–*PpB‐GATA4*) through mutant analysis and revealed their critical role in N‐responsive growth. We found that mutants of the moss *B‐GATA* genes are defective in N‐regulated protonema growth and in N‐regulated gene expression. Building on these findings, we extended our analysis to *Arabidopsis* mutants of the *B‐GATAs*, where we also uncovered defects in N‐responsive growth and transcription regulation. Taken together, our results highlight an evolutionarily conserved function for B‐GATA transcription factors in plant growth in response to N availability.

## Materials and Methods

### Biological material

*Physcomitrium patens* (Hedw.) Mitt. ecotype Reute (Hiss *et al*., 2017) and *Arabidopsis thaliana* (L.) *Heynh*. ecotype Columbia (Col‐0) were used as wild‐type controls in all experiments. The following *Arabidopsis mutants* were previously characterized: *gnc gnl* (R. Richter *et al*., 2010) and *gnc gnl gata15 gata16 gata17 gata17l* (referred to as *gata hex*) (Schroder *et al*., 2023). *Physcomitrium* mutants were generated as part of this study, as described below.

Methods and primers used in this study are provided as supplemental data (Supporting Information Methods S1; Table S1).

## Results

### *Physcomitrium patens* encodes four B‐GATA transcription factors

**Fig. 1:**
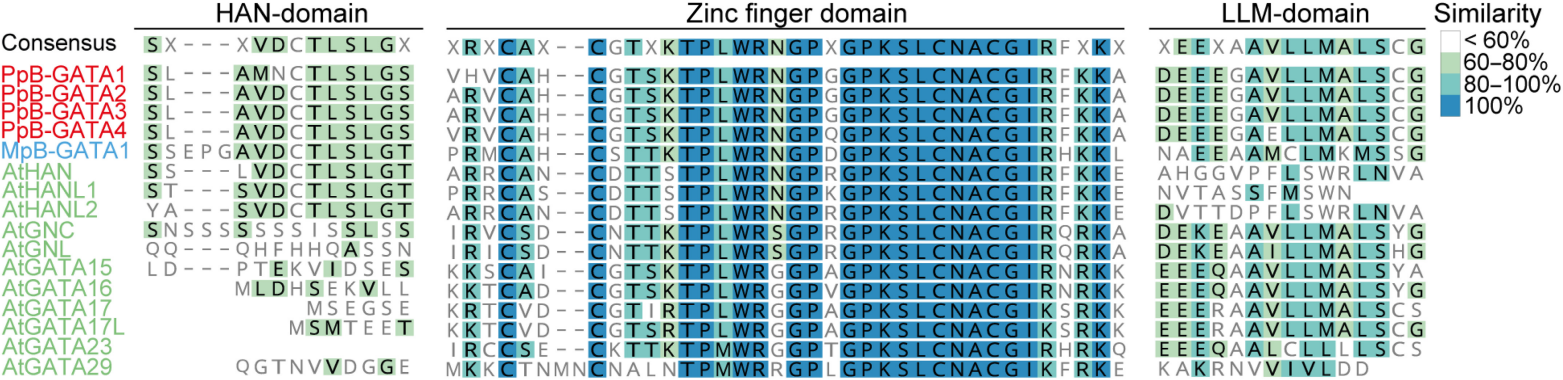
Sequence comparison of B‐GATA factors from *Physcomitrium patens* (Pp), *Marchantia polymorpha* (Mp), and *Arabidopsis thaliana* (At). Alignment of the conserved HAN‐domain, zinc finger domain, and LLM‐domain from selected B‐GATA *trans*cription factors of *Physcomitrium patens* (Pp), *Marchantia polymorpha* (Mp), and (At). Residue similarities and identities are color‐coded according to the scheme shown in the figure legend.

In angiosperms, B‐class GATAs are divided into those containing either a HAN‐ or LLM‐domain. By contrast, the single B‐GATA from the liverwort *Marchantia* and all four B‐GATAs from the moss *Physcomitrium* possess both HAN‐ and LLM‐domains, suggesting that the ancestral land plant B‐GATA may have harbored both domains (Figs 1, S1) (Schwechheimer *et al*., 2022; Schroder *et al*., 2023). Phylogenetic analysis shows that the four *Physcomitrium* B‐GATAs, which we have designated PpB‐GATA1 to PpB‐GATA4, are more closely related to each other than to B‐GATAs from *Marchantia* or *Arabidopsis*, indicating that this gene family expanded after the divergence of mosses from the last common ancestor of mosses and liverworts (Fig. S1).

Gene expression data from public repositories revealed that all *PpB‐GATAs* are broadly expressed across diverse tissues. Among them, *PpB‐GATA1* and *PpB‐GATA3* showed the widest and highest expression, particularly in vegetative tissues such as chloronema, caulonema, gametophores, and rhizoids (Fig. S2) (Winter *et al*., 2007; Ortiz‐Ramirez *et al*., 2016). *PpB‐GATA2* was most strongly expressed in chloronema and rhizoids, with lower levels in caulonema and gametophores, whereas *PpB‐GATA4* exhibited strong expression primarily in mature sporophytes and minimal expression in vegetative tissues (Fig. S2) (Winter *et al*., 2007; Ortiz‐Ramirez *et al*., 2016). These data point to *PpB‐GATA1* and *PpB‐GATA3* as the most broadly and strongly expressed genes within this family.

### Generation and molecular characterization of *Ppb‐gata* mutants

To investigate the biological roles of the *PpB‐GATAs*, we employed CRISPR/Cas9‐based genome editing to target the four genes (Fig. S3). Following transformation, mutagenesis, and PCR‐based genotyping, we isolated single, double, and triple mutants of *PpB‐GATA1*, *PpB‐GATA3*, and *PpB‐GATA4*. These included single mutant alleles (*Ppb‐gata1‐1*, *Ppb‐gata3‐1*, *Ppb‐gata4‐1*, *Ppb‐gata4‐2*), the double mutant *Ppb‐gata3‐2/4‐3*, and the two triple mutants *Ppb‐gata1‐1/3‐2/4‐3* and *Ppb‐gata1‐3/3‐2/4‐3*, all of which harbored frameshift‐inducing deletions or insertions and were consequently classified as loss‐of‐function mutants (Fig. S3). An exception was the *Ppb‐gata1‐2* allele in the *Ppb‐gata1‐2/4‐2* double mutant, which contained a 15 bp duplication resulting in an in‐frame insertion of six nonnative amino acids (Q‐S‐T‐T‐Q‐C) at the edited site (Fig. S3).

Whole‐genome sequencing confirmed the mutations and allowed assessment of background variation (Table S2). The *Ppb‐gata1‐1* mutant exhibited several polymorphisms in noncoding regions, with two missense mutations found in the genes *Pp3c6_23550*, encoding a pentatricopeptide repeat protein, and *Pp3c10_15160*, encoding a CORYNE‐related receptor‐like kinase gene. Due to the lack of an alternative *Ppb‐gata1* allele, we proceeded with this line despite these background mutations. In the originally analyzed *Ppb‐gata4‐1* allele, sequencing also revealed a possible but ultimately unresolvable *Ppb‐gata3* mutation, prompting us to use the alternative allele *Ppb‐gata4‐2* for further experiments. Among the triple mutants, *Ppb‐gata1‐1/3‐2/4‐3* harbored 23 insertions or deletions and 15 single nucleotide polymorphisms, whereas *Ppb‐gata1‐3/3‐2/4‐3* had no detectable background mutations and was therefore selected for further analysis (Table S2). For clarity, the allele nomenclature in the remaining manuscript is reduced to the gene mutant names, omitting the allele specifications.

Despite multiple attempts and extensive genotyping, we were unable to obtain *Ppb‐gata2* mutants by CRISPR/Cas9 mutagenesis or by homologous recombination.

### *PpB‐GATAs* are required for protonema growth and pigment biosynthesis

**Fig. 2:**
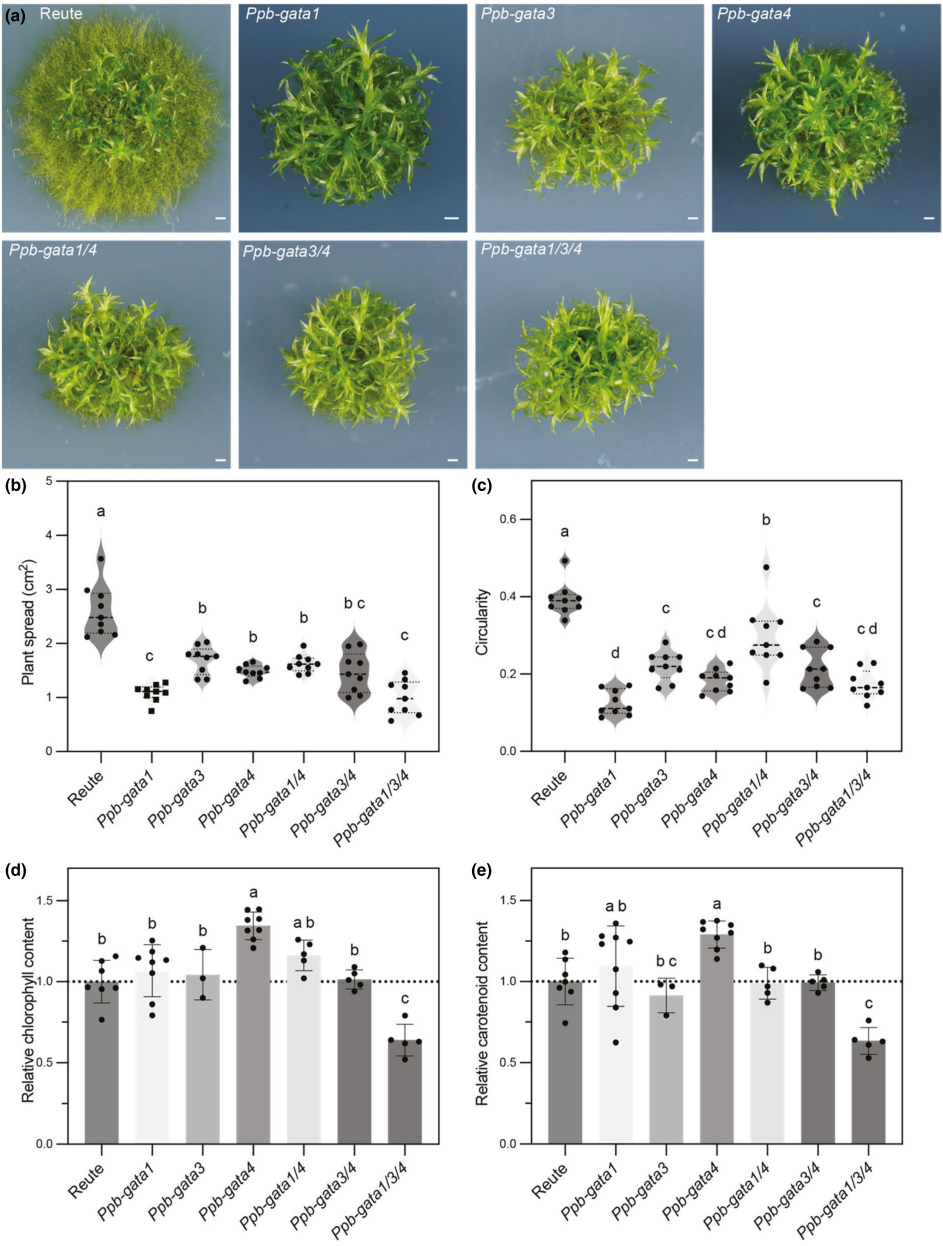
Characterization of *Physcomitium patens Ppb‐gata* mutant phenotypes. (a) Representative images of 4‐wk‐old *Physcomitrium* plants of the indicated genotypes. Bar, 1 mm. (b, c) Quantification of plant morphology based on the images shown in (a). (b) Spread area of the plants. (c) Circularity index as a measure of shape compactness. Each data point represents an independent biological replicate; horizontal black lines indicate the median, and dotted lines mark the first (Q1) and third (Q3) quartiles. Different letters indicate statistically significant differences determined by Brown–Forsythe and Welch ANOVA followed by Dunnett's T3 multiple comparisons test (b) or by one‐way ANOVA with Tukey's multiple comparisons test (c). (d, e) Quantification of photosynthetic pigments in the indicated genotypes: (d) total Chl content (Chl*a* + Chl*b*); (e) total carotenoid content, both relative to the concentrations detected in the Reute wild‐type. Each data point represents an independent biological replicate; bars show mean ± SD. Different letters indicate statistically significant differences as determined by one‐way ANOVA followed by Tukey's multiple comparisons test.

All *Ppb‐gata* mutants exhibited a pronounced reduction in plant spread when grown on medium containing both NH_4_^+^ and NO_3_^−^ as N sources (Fig. 2a,b). This phenotype was attributed to impaired protonema growth, which also led to a less circular plant morphology, reflected in a decreased circularity index (Fig. 2).

Given that mutants of *B‐GATAs* in *Arabidopsis* and *Marchantia* show defects in Chl and carotenoid biosynthesis, we measured pigment levels in the *Physcomitrium* mutants (Ranftl *et al*., 2016; Bastakis *et al*., 2018; Schroder *et al*., 2023). The *Ppb‐gata1/3/4* triple mutant showed a significant reduction in both Chl and carotenoids compared with the Reute wild‐type strain, while the other mutants had levels comparable to wild‐type (Fig 2d,e). Our results indicate that the *Physcomitrium* B‐GATAs defective in the *Ppb‐gata1/3/4* triple mutant, like their orthologs from *Arabidopsis* and *Marchantia*, are important for proper Chl and carotenoid biosynthesis.

### *Ppb‐gata* mutants are defective in protonema formation

**Fig. 3:**
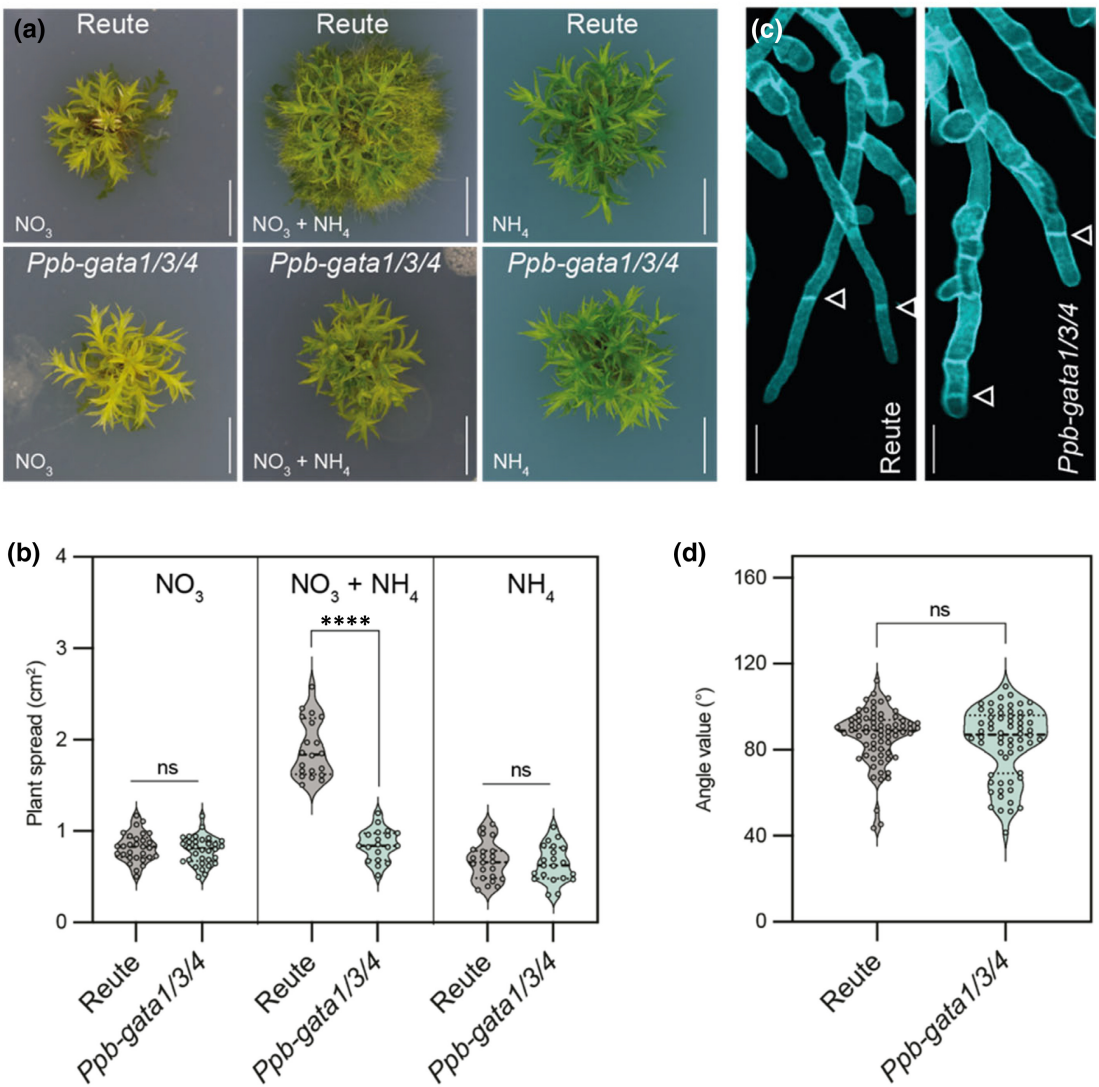
Loss of *B‐GATAs* influences N‐dependent growth responses in *Physcomitrium patens*. (a) Representative images of 4‐wk‐old *Physcomitrium* Reute (wild‐type) and *Ppb‐gata1/3/4* triple mutant plants grown on media containing the indicated N sources. Bar, 5 mm. (b) Quantification of plant spread area from the plants shown in (a). Each data point represents an independent biological replicate; horizontal black lines indicate the median, and dotted lines mark the first (Q1) and third (Q3) quartiles. Unpaired *t*‐test, with Welch's correction applied when variances were significantly different: ****, *P* < 0.0001; ns, not significant. (c) Representative confocal images of calcofluor white–stained protonemal filaments. Arrows mark the division planes used for angle measurements in (d). Bar, 50 μm. (d) Quantification of cell division plane angles from the cells indicated in (c). Each data point represents an independent biological replicate; horizontal black lines indicate the median, and dotted lines mark the first (Q1) and third (Q3) quartiles. Unpaired *t*‐test: ns, not significant.

The reduced size of *Ppb‐gata* mutants was primarily due to a strong impairment in protonema formation compared with the wild‐type (Fig. 2a). In *Physcomitrium*, protonema development is induced by the combined presence of nitrate (NO_3_^−^) and ammonium (NH_4_^+^) as N sources (Perroud *et al*., 2018). When grown on media supplemented with NO_3_^−^ and NH_4_^+^, wild‐type plants exhibited increased plant spread and protonema formation, whereas these responses were absent in the *Ppb‐gata1/3/4* triple mutant (Fig. 3a,b). As reported previously, the presence of only NO_3_^−^ or NH_4_^+^ as a single N source was insufficient to induce protonema development in either wild‐type or mutant plants (Perroud *et al*., 2018).

Protonema tissue consists of two cell types: chloronema and caulonema, which can be distinguished by the angles between adjoining cell walls (Coudert *et al*., 2019). Analysis of cell wall angles revealed no significant differences in chloronema and caulonema proportions between wild‐type and the *Ppb‐gata1/3/4* triple mutant (Fig. 3c–d). We therefore conclude that the triple mutant is specifically impaired in N‐induced protonema formation but not in the differentiation of the chloronema and caulonema cell types.

### *PpB‐GATA1* overexpression produces opposite phenotypes to loss‐of‐function mutants

To further assess the role of *PpB‐GATAs*, we generated overexpression lines of *PpB‐GATA1*, one of the highly expressed family members (Fig. S4). In three independent lines, RT‐qPCR confirmed a 2‐ to 7‐fold increase in *PpB‐GATA1* expression (Fig. S4a). These overexpression lines exhibited significantly increased levels of Chl and carotenoids, which is in contrast to the reduced pigment content of the *Ppb‐gata1/3/4* triple mutant (Fig. S4b,c).

Interestingly, all overexpressors were smaller than the wild‐type, regardless of whether plants were grown on NO_3_^−^ and NH_4_^+^ or on NO_3_^−^ alone. Moreover, the extent of size reduction correlated with transgene expression levels (Fig. S4d,e). Overexpression lines also showed increased circularity under both N regimes, a parameter that correlates with enhanced protonema development (Fig. S4f–i). The increased protonemal circularity observed even in NO_3_^−^‐only conditions suggests that PpB‐GATAs can promote protonema development independently of NH_4_^+^ availability.

### *Ppb‐gata* mutants accumulate cytokinin

**Fig. 4:**
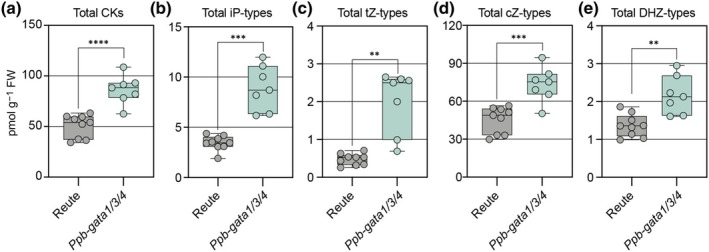
Loss of B‐GATAs influences endogenous cytokinin levels in *Physcomitrium patens*. (a–e) Quantification of cytokinin (CK) metabolites in 4‐wk‐old *Physcomitrium* Reute (wild‐type) and *Ppb‐gata1/3/4* triple mutant plants. Each graph represents the concentration of a specific cytokinin form or metabolite as indicated. iP, isopentenyladenine; tZ, *trans*‐zeatin; cZ, *cis*‐zeatin; DHZ, dihydrozeatin. Each data point represents an independent biological replicate (*n* = 3–5); the whiskers indicate the full data range (minimum to maximum), boxes show the interquartile range (IQR; spanning the 25^th^ to 75^th^ percentiles), and the central line indicates the median value of the data distribution. Unpaired *t‐*test, with Welch's correction applied when variances were significantly different: **, *P* < 0.01; ***, *P* < 0.001; ****, *P* < 0.0001.

A previous study showed that elevated cytokinin levels suppress plant spread in *Physcomitrium* (Coudert *et al*., 2019). To examine whether the *Ppb‐gata1/3/4* triple mutant phenotype could potentially be linked to cytokinin biosynthesis, we quantified cytokinin levels in wild‐type and mutant moss. This quantification revealed significantly higher cytokinin levels in the *Ppb‐gata1/3/4* triple mutant compared with the wild‐type, and consequently, increased cytokinin levels may contribute to the growth defect observed in the mutants (Fig. 4; Table S3).

### Nitrogen metabolism and uptake are not impaired in the *Ppb‐gata* triple mutant

**Fig. 5:**
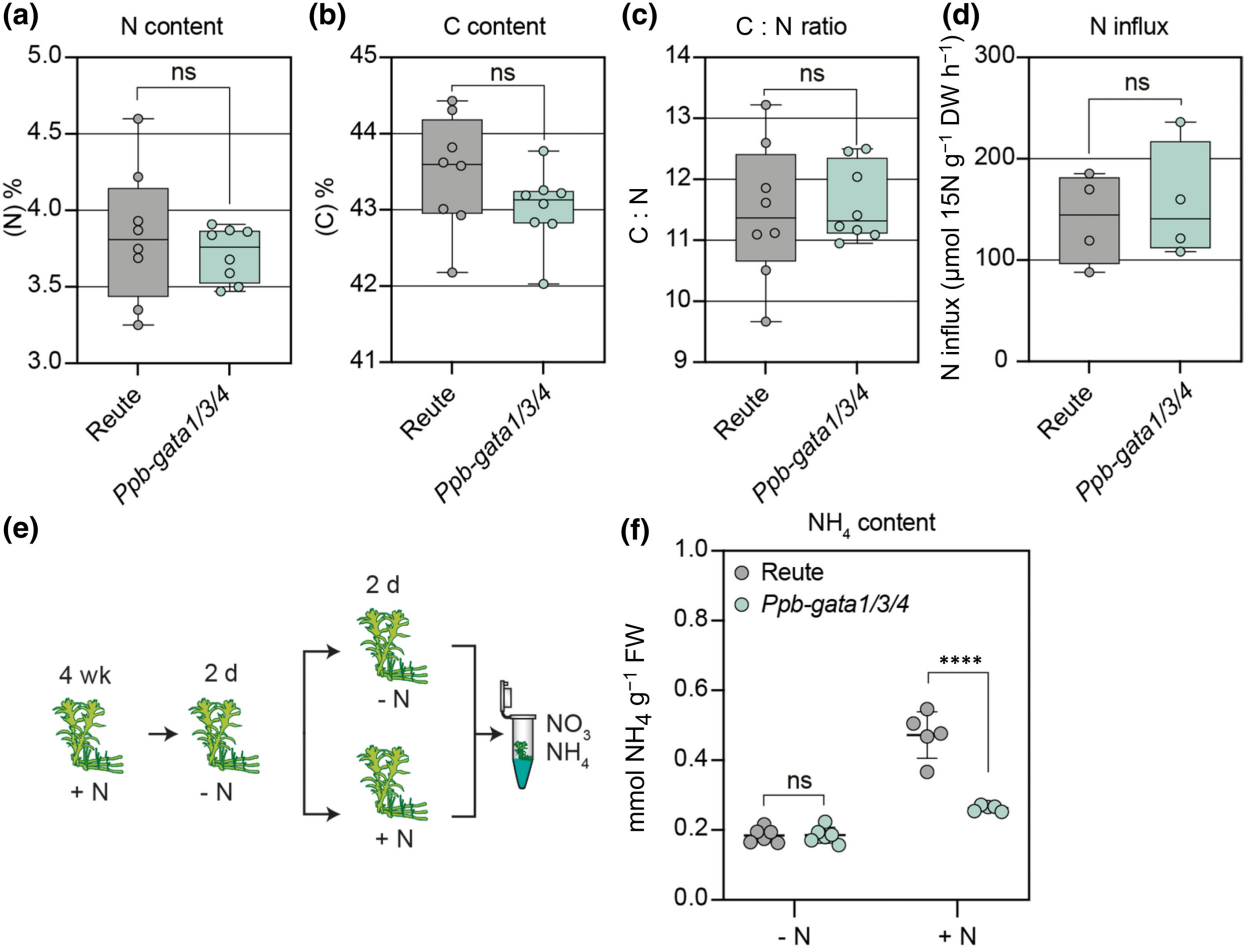
Loss of *B‐GATA* transcription factors does not affect endogenous nitrogen (N) and carbon (C) levels in *Physcomitrium patens*. (a–c) Quantification of total N (a), total C (b), and the C : N ratio (C) in 6‐d‐old *Physcomitrium* wild‐type and *Ppb‐gata1/3/4* mutant protonemata grown on N‐containing medium. Each data point represents an independent biological replicate (*n* = 3–5 colonies); the whiskers indicate the full data range (minimum to maximum), boxes show the interquartile range (IQR; spanning the 25^th^ to 75^th^ percentiles), and the central line indicates the median value of the data distribution. Unpaired *t*‐test: ns, not significant. (d) N influx rates measured in 13‐d‐old wild‐type and *Ppb‐gata1/3/4* mutant plants following a 12 min exposure with 10 mM ^15^N‐labeled KNO_3_ and ^15^N‐labeled NH_4_^+^. Each data point represents an independent biological replicate (*n* = 4 colonies); the whiskers indicate the full data range (minimum to maximum), boxes show the interquartile range (IQR; spanning the 25^th^–75^th^ percentiles), and the central line indicates the median value of the data distribution. Unpaired *t*‐test: ns, not significant. (e) Schematic overview of the N metabolism experiment. Plants were first N‐starved for 48 h, then transferred to either N‐containing medium (+N; 10 mM KNO_3_ and 5 mM (NH_4_)_2_C_4_H_4_O_6_) or a mock medium lacking nitrogen (‐N; 10 mM KCl). (f) Ammonium (NH_4_^+^) concentrations in tissue extracts measured after N resupply. Each data point represents an independent biological replicate (*n* = 4 colonies); bars indicate mean ± SD. Two‐way ANOVA followed by Šídák's multiple comparisons test; ****, *P* < 0.0001; ns, not significant. A corresponding measurement of tissue nitrate (NO_3_^−^) content yielded levels below the detection limit.

To determine whether the reduced growth in the *Ppb‐gata1/3/4* mutant was due to altered N metabolism or a disrupted C : N (carbon‐to‐nitrogen) balance, we conducted elemental analysis on 4‐wk‐old plants grown on standard media (10 mM KNO_3_, 5 mM (NH_4_)_2_C_4_H_4_O_6_). No significant differences in total N or C content, nor in their ratio, were detected between wild‐type and mutant plants, suggesting that N and C metabolism were not impaired in the mutant tissue analyzed (Fig. 5a–c).

We next tested whether N uptake capacity differed between genotypes. Plants were N‐starved for 2 d and then exposed to ^15^N‐labeled NH_4_NO_3_ for 12 min. Isotope analysis revealed no significant differences in ^15^N incorporation between wild‐type and mutant plants, indicating that N uptake was not impaired in the *Ppb‐gata1/3/4* mutant in the time frame observed in the study (Fig. 5d).

To examine internal N metabolism, plants starved of N for 2 d were transferred either to N‐containing media (10 mM KNO_3_, 5 mM (NH_4_)_2_C_4_H_4_O_6_) or to N‐free control media. After 2 d, NO_3_^−^ levels were below the detection limit in both genotypes, suggesting rapid NO_3_^−^ assimilation. By contrast, NH_4_^+^ levels were significantly lower in the *Ppb‐gata1/3/4* mutant compared with the wild‐type (Fig. 5e,f). Since our N uptake study had shown that N uptake may not be affected in the mutant, the reduced NH_4_^+^ content in the mutant likely reflects increased NH_4_^+^ assimilation or altered N metabolism downstream of uptake.

### *Ppb‐gata* mutants display severely impaired transcriptional responses to N supply

**Fig. 6:**
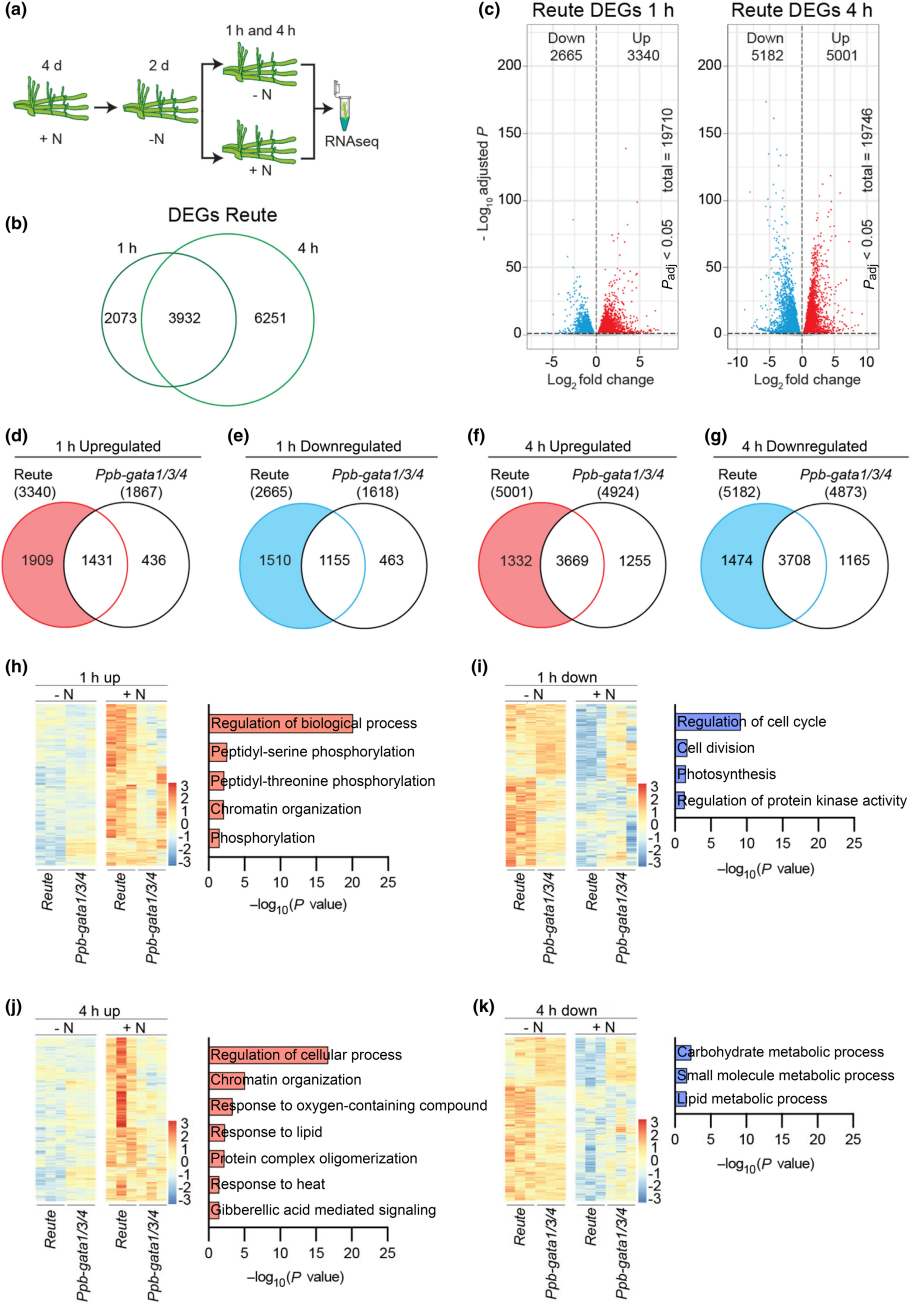
Transcriptomic characterization of the N response in *Physcomitrium patens* and the *Ppb‐gata1/3/4* mutant. (a) Schematic overview of the RNA‐seq experiment. Wild‐type and *Ppb‐gata1/3/4* mutant protonemata were pre‐cultured on N‐containing medium, starved of N for 2 d, and subsequently transferred to either N‐containing (+N; 10 mM KNO_3_ and 5 mM (NH_4_)_2_C_4_H_4_O_6_) or N‐free mock (–N; 10 mM KCl) medium for 1 or 4 h before RNA extraction. (b) Venn diagram showing overlap of differentially expressed genes (DEGs) between +N and –N treatments in wild‐type *Physcomitrium* (Reute strain) at the 1‐ and 4‐h time points. (c) Volcano plots illustrating the distribution of DEGs in the wild‐type at the 1‐ and 4‐h time points, highlighting genes significantly upregulated or downregulated in response to N. The total number of variables (genes) is provided. (d–g) Venn diagrams comparing DEGs between the wild‐type and the *Ppb‐gata1/3/4* mutant under +N vs –N conditions, separated into upregulated (d, f) and downregulated (e, g) gene sets at 1 h (d, e) and 4 h (f, g). Color‐coded sections indicate DEGs exclusive to the wild‐type, which were used for subsequent GO enrichment analysis. (h–k) Heatmaps and corresponding gene ontology (GO) enrichment analyses of DEGs that were regulated in response to N in the wild‐type but not in the *Ppb‐gata1/3/4* mutant, potentially representing PpB‐GATA‐dependent *trans*cripts. Panels show upregulated (h, j) and downregulated (i, k) gene sets at 1 h (h, i) and 4 h (j, k).

To assess whether the *Ppb‐gata1/3/4* exhibited altered transcriptional responses to N availability, we performed RNA‐seq analysis on 6‐d‐old wild‐type and *Ppb‐gata1/3/4* mutant plants. To this end, plants were first grown on N‐containing medium (10 mM KNO_3_, 5 mM (NH_4_)_2_C_4_H_4_O_6_) for 2 d, then transferred to N‐free medium for 2 d, followed by re‐exposure to either the N‐containing medium (10 mM KNO_3_, 5 mM (NH_4_)_2_C_4_H_4_O_6_) or 10 mM KCl mock control medium for 1 or 4 h, taking into account the presence of 10 mM potassium (K) in the N‐containing medium (Fig. 6a).

Principal component analysis (PCA) of samples from both time points revealed that N treatment had a strong effect on global gene expression, whereas the variation between genotypes was comparatively smaller (Fig. S5). One *Ppb‐gata1/3/4* replicate from the 1‐h time point showed a distinct clustering pattern compared with the other two replicates. However, neither the 1.5 interquartile range (IQR) rule nor Mahalanobis distance analysis (χ^2^(2), *P* < 0.01) identified this replicate as a statistical outlier, and it was therefore retained for downstream analyses (Mahalanobis, 1936; Tukey, 1977).

In the wild‐type, DESeq2 identified 6005 differentially expressed genes (DEGs) after 1 h of N treatment and 10 183 DEGs after 4 h (Fig. 6b,c; Table S4). Of these, 3932 DEGs were differentially regulated at both time points, indicating a sustained transcriptional response to N availability. Gene ontology (GO) analysis showed that N‐regulated DEGs were enriched for GO terms related to biological regulation, biosynthetic processes, and cell cycle control, likely reflecting the stimulatory effect of N on cellular metabolism and growth in *Physcomitrium* (Fig. S6; Table S5).

By contrast, the *Ppb‐gata1/3/4* mutant failed to regulate a substantial portion of these N‐responsive DEGs. Specifically, 1909 (57%) of the 3340 upregulated DEGs and 1510 (57%) of the 2665 downregulated DEGs identified in the wild‐type after 1 h of N treatment were not differentially expressed in the mutant (Fig. 6d,e; Table S4c). At the 4‐h time point, 1332 (27%) of the 5001 upregulated DEGs and 1474 (28%) of the 5182 downregulated DEGs in the wild‐type were not responsive in the mutant. These results indicate that N‐responsive transcriptional responses are strongly dependent on the presence of the *PpB‐GATA* genes.

GO enrichment of the PpB‐GATA–dependent gene sets further revealed enrichment in categories such as regulation of biological processes, cellular function, cell cycle progression, and cell division (Fig. 6h–k; Table S6). Notably, orthologs of the *Arabidopsis* ethylene receptor genes *ETR1* (Pp6c14_10160; orthologous to AT1G66340) and *ETR2* (Pp6c9_9400; AT3G23150), both two‐component sensor histidine kinases, were upregulated by N treatment in the wild‐type but unresponsive in the *Ppb‐gata1/3/4* mutant. Similarly, 10 Ca^2+^‐dependent protein kinase genes, several *AUXIN RESPONSE FACTORs* (*ARFs*), including Pp6c14_9370 (AT2G46530, *ARF11*), Pp6c4_6680 (AT1G19850, *ARF5*), Pp6c5_4850 (AT1G59750, *ARF1*), and Pp6c2_14160 (AT1G19220, *ARF19*), as well as three SWI/SNF chromatin remodeling factors were differentially expressed in response to N in the wild‐type, but not in the mutant. Finally, the N‐regulation of six *XYLOGLUCAN ENDOTRANSGLYCOSYLASE* (*XET*) genes, genes previously linked to protonema differentiation following N treatment (Perroud *et al*., 2018), was disrupted in the *Ppb‐gata1/3/4* mutant, further supporting a critical role for B‐GATA transcription factors in mediating N‐responsive developmental transitions.

To examine the transcription regulation of genes with a proposed role in N uptake and transport, metabolism, and relevant transcriptional responses, we generated a list of *Physcomitrium* orthologues using a combination of Orthofinder and Diamond BLAST of genes with a role in these processes that had been previously identified in *Arabidopsis* (Fig. S7; Table S7a) (Vidal *et al*., 2020). Among the orthologous genes encoding transporter genes, we detected enhanced N regulation of the high‐affinity nitrate transporter orthologue *PpNRT2.1* and a loss of N regulation of the ammonium transporter orthologue *PpAMT1* and the nitrate transporter orthologues *PpNPF6.2*, *PpNPF8.1/8.4*, and *PpNPF8.3* (Fig. S7a). Among the N metabolism genes, we detected a loss or weakened N regulation in the *Ppb‐gata1/3/4* mutant of the amino acid transporter orthologue *PpAAT1* and the glutamate decarboxylase *PpGAD3*, but stronger N regulation of two orthologues of the nitrate reductase PpNIA1 and of the glutamine synthetases *PpGS2* and *PpGSR2* (Fig. S7b). Several genes orthologous to transcription factors with a demonstrated or proposed role in N responses showed differential expression between the wild‐type and the mutant, including *PpCDF1*, *PpLBD37/PpLBD38*, *PpNLP6*, *PpNLP7*, as well as *PpTCP20* (Fig. S7c). The *MKN2* (*KNOX2*) gene, Pp6c25_4720, previously associated with a phenotype similar to that observed in *Ppb‐gata1/3/4*, was not differentially expressed in the mutant, indicating that its misexpression is unlikely to account for the *GATA* gene mutant phenotype (Coudert *et al*., 2019).

Finally, we analyzed whether *PpB‐GATA* gene expression was regulated by N under our experimental conditions (Fig. S7d). Specifically, at the 4 h time point, we found that three *PpB‐GATA* genes were upregulated in response to the N treatment, while *PpB‐GATA4* expression was downregulated. Accordingly, *PpB‐GATA1*, *PpB‐GATA2*, and *PpB‐GATA3* behave like *Arabidopsis GNC*, and all four *PpB‐GATAs* could be classified as N‐regulated *B‐GATA* genes.

### Expression of biosynthesis genes may explain the Chl and cytokinin phenotypes of *Ppb‐gata1/3/4*

To understand whether the differences in Chl accumulation could be explained through the available RNA‐seq data, we generated a list of *Physcomitrium* genes orthologous to genes that had been implicated in Chl and cytokinin biosynthesis in *Arabidopsis* using OrthoFinder and/or Diamond BLAST (Table S7b,c) (Emms & Kelly, 2019).

With regard to the expression of Chl biosynthesis genes, we identified many genes contributing to Chl biosynthesis, for example genes orthologous to the heme biosynthesis genes *HEME*, *HEMEF*, and *HEMEG*, as well as *MAGNESIUM CHELATASE SUBUNIT I* (*CHLI*) and *PROTOCHLOROPHYLLIDE OXIDOREDUCTASE A* (*PORA*) as being downregulated in *Ppb‐gata1/3/4* in comparison with the Reute wild‐type (Fig. S8). The decreased expression of these genes may explain the reduced Chl biosynthesis observed in the mutants.

To establish a link to cytokinin biology, in view of the altered cytokinin levels observed in the mutants, we retrieved a list of genes with a proposed role in cytokinin biosynthesis (*trans*‐zeatin; tZ) from *Physcomitrium* from the KEGG database, and identified orthologues of genes related to cytokinin signaling in the literature and using a combination of Orthofinder and/or Diamond BLAST (Table S7c) (Pils & Heyl, 2009; Kieber & Schaller, 2014; Hata *et al*., 2025). Several genes encoding for isopentenyltransferases (*IPT*), the *LONELY GUY* (*LOG*) cytokinin nucleoside 5′‐monophosphate phosphoribohydrolases, and the catabolic enzyme cytokinin oxidase/dehydrogenase (*CKX*) were more strongly expressed in *Ppb‐gata1/3/4* in comparison with the wild‐type, providing a possible explanation for the abundance of cytokinin in *Ppb‐gata1/3/4* (Fig. S9). We further noted the strong upregulation of two genes encoding for cytokinin receptors, *PpCHK2* (Pp6c16_5950) and *PpCHK3* (Pp6c6_3870).

### *Arabidopsis* mutants of LLM‐domain B‐GATAs exhibit reduced growth responses to nitrogen supply

**Fig. 7:**
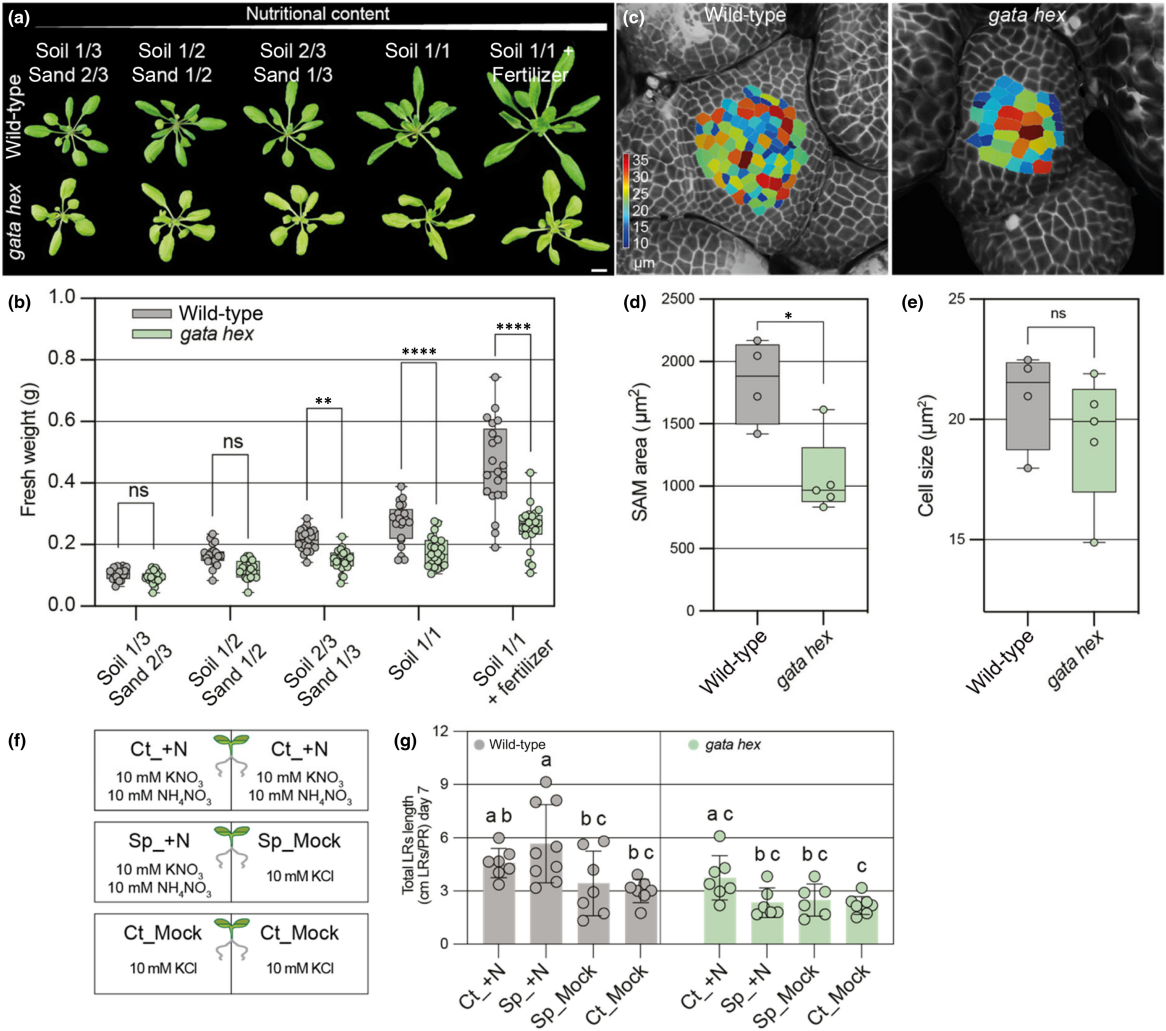
Loss of LLM‐domain B‐GATAs impairs N‐dependent growth responses in *Arabidopsis thaliana*. (a) Representative images of 4‐wk‐old *Arabidopsis* wild‐type (Col‐0) and *gata hexuple* (*gata hex*) mutant plants, grown under varying soil/sand mixtures or soil supplemented with fertilizer. Bar, 1 cm. (b) Quantification of fresh weight in *Arabidopsis* plants as shown in (a). Each data point represents an independent biological replicate (one entire rosette); the whiskers indicate the full data range (minimum to maximum), boxes show the interquartile range (IQR; spanning the 25^th^–75^th^ percentiles), and the central line indicates the median value of the data distribution. Two‐way ANOVA comparisons test, followed by Šídák's multiple comparisons performed for each soil and sand combination. **, *P* < 0.01; ****, *P* < 0.0001, ns, not significant. (c) Representative confocal images of shoot apical meristems (SAMs) from 4‐wk‐old soil‐grown plants, reconstructed using Morphographics X. (d, e) Quantification of SAM surface area (d) and average cell size (e) from the samples shown in (c). Each data point represents an independent biological replicate (one inflorescence meristem); the whiskers indicate the full data range (minimum to maximum), boxes show the interquartile range (IQR; spanning the 25^th^–75^th^ percentiles), and the central line indicates the median value of the data distribution. Unpaired *t*‐test: *, *P* < 0.05; ns, not significant. (f) Schematic representation of the split‐root setup used for the analysis shown in (g). Roots were subjected to three conditions: Ct_ +N (control + nitrogen) and Ct_Mock (control mock), where both sides of the root system were exposed to either + N or mock treatment, respectively; and Split_ + N/Sp_Mock, where one side was supplied with N and the other with mock (KCl). (g) Total lateral root (LR) growth in wild‐type Col‐0 and *gata hex* under the conditions described in (f). Total LR length was normalized to the primary root (PR) length. Each point represents an independent biological replicate; bars indicate mean ± SD. Different letters denote statistically significant differences (two‐way ANOVA followed by Tukey's multiple comparisons test).

The observation of a differential N response in *Physcomitrium* prompted us to investigate whether *Arabidopsis* mutants of *B‐GATA* genes also show altered growth responses to N availability. To this end, we compared the wild‐type with two previously characterized mutant lines: *gnc gnl*, which carries mutations in the LLM‐domain B‐GATA genes *GNC* and *GNL*, and *gata hex*, which is deficient in all six *LLM‐domain B‐GATA* genes, including *GNC* and *GNL* (R. Richter *et al*., 2010; Schroder *et al*., 2023). Plants were grown on mixtures of N‐poor sand and N‐rich soil, following established protocols (S. Richter *et al*., 2010; Landrein *et al*., 2018; Schroder *et al*., 2023). To prevent N availability from affecting germination, seeds were first germinated in full‐strength soil and grown for 1 wk before being transferred to the respective soil/sand mixtures for an additional 4 wk. In these experiments, *gata hex* mutants displayed visibly reduced rosette diameters when grown on soil alone or on fertilized soil, whereas these differences were not apparent on sand‐enriched substrates (Figs 7a, S10a,b). Fresh weight measurements after 4 wk revealed significant growth reduction in *gata hex* on 2 : 1 soil‐to‐sand mixtures, with a milder reduction also observed in *gnc gnl* mutants (Figs 7b, S10a,b). The pronounced increase in fresh weight across all genotypes following fertilization suggests that N, or potentially other macro‐ or micronutrients present in the fertilizer, is a limiting factor for growth in our standard soil conditions.

To further assess the impact of *B‐GATA* deficiency on N‐responsive growth, we examined shoot apical meristem (SAM) morphology in soil‐grown wild‐type and *gata hex* plants. SAM size, but not cell size, was significantly reduced in the mutant, supporting the notion that N supply cannot be effectively translated into enhanced plant vigor in the absence of functional B‐GATA transcription factors (Fig. 7c–e).

To investigate the role of B‐GATAs in N‐related systemic responses, we performed a split‐root assay (Figs 7f, S10c) (Ruffel *et al*., 2011). This approach enables the two root systems of a single plant to be exposed to distinct nutritional regimes, thereby generating heterogeneous nitrogen conditions. Root architecture was analyzed 7 d after *trans*fer under three conditions: (Ct_ +N) a homogeneous N‐rich environment with both sides containing 10 mM KNO_3_ + 10 mM NH_4_NO_3_, (Ct_Mock) a homogeneous N‐depleted environment with both sides containing 10 mM KCl, and (Sp_ +N/Sp_Mock) a heterogeneous split environment with one side containing 10 mM KNO_3_ + 10 mM NH_4_NO_3_ and the other side containing 10 mM KCl. In the wild‐type plants, we observed a compensatory lateral root growth in the heterogeneous condition, with preferential lateral root elongation on the nitrogen‐supplemented side (Sp_ +N) compared with the nitrogen‐free side (Sp_Mock) (Figs 7g, S10d). This compensatory response was severely compromised in the *gata hex* mutant but still preserved in *gnc gnl* (Figs 7g, S10d,e). In contrast to previous reports, lateral root growth in N‐depleted conditions (Ct_Mock) did not surpass growth under N‐sufficient conditions (Ct_ +N) in the wild‐type in our conditions, which may be explained by the fact that we used different N treatments for our experiment than those used in the previous report to stay in the context of the other experimental setups used for our studies (Fig. 7g).

### *Arabidopsis gata hex* mutants have elevated cytokinin levels

**Fig. 8:**
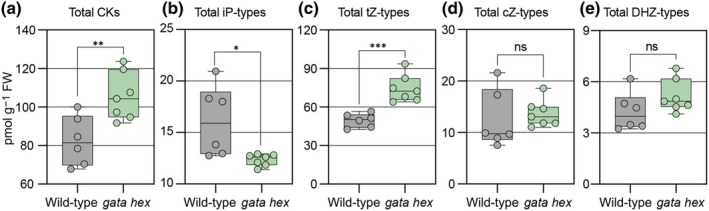
Loss of LLM‐domain B‐GATA transcription factors alters endogenous cytokinin levels in *Arabidopsis thaliana*. (a–e) Quantification of cytokinin (CK) metabolites in 4‐wk‐old *Arabidopsis* wild‐type (Col‐0) and *gata hexuple* (*gata hex*) mutant plants. iP, isopentenyladenine; tZ, *trans*‐zeatin; cZ, *cis*‐zeatin; DHZ, dihydrozeatin. Each data point represents an independent biological replicate (*n* = 2/3 rosettes); the whiskers indicate the full data range (minimum to maximum), boxes show the interquartile range (IQR; spanning the 25^th^–75^th^ percentiles), and the central line indicates the median value of the data distribution. Unpaired *t*‐test, with Welch's correction applied when variances were significantly different: *, *P* < 0.05; **, *P* < 0.01; ***, *P* < 0.001; ns, not significant.

Vegetative growth in response to N is mediated by cytokinin biosynthesis and signaling (Landrein *et al*., 2018). We therefore also quantified cytokinin levels in 4‐wk‐old rosettes of wild‐type and *gata hex* mutants. The analysis revealed a significant increase in *trans*‐zeatin (tZ)‐type cytokinins in *gata hex*, along with comparatively minor decreases in isopentenyladenine (iP)‐type cytokinins (Fig. 8a–e). As tZ‐type cytokinins contribute more substantially to the total cytokinin content, this shift resulted in an overall increase of total cytokinins in *gata hex* when compared with the wild‐type (Fig. 8a). Despite these elevated cytokinin levels, the *gata hex* mutants had reduced shoot growth and a smaller shoot apical meristem (SAM) area (Fig. 7). This indicates that the growth defect is not due to a deficiency in cytokinin synthesis but rather suggests that the lack of the *B‐GATA* may disrupt the plant's ability to respond to cytokinin signaling. Further, since the systemic N response in the split‐root assay is cytokinin‐dependent, but *gata hex* exhibits reduced lateral root proliferation despite increased cytokinin levels (Fig. 7g), the *Arabidopsis* B‐GATAs may function as mediators downstream from cytokinins in systemic N signaling.

### Arabidopsis *gata hex* mutants have increased nitrogen levels

**Fig. 9:**
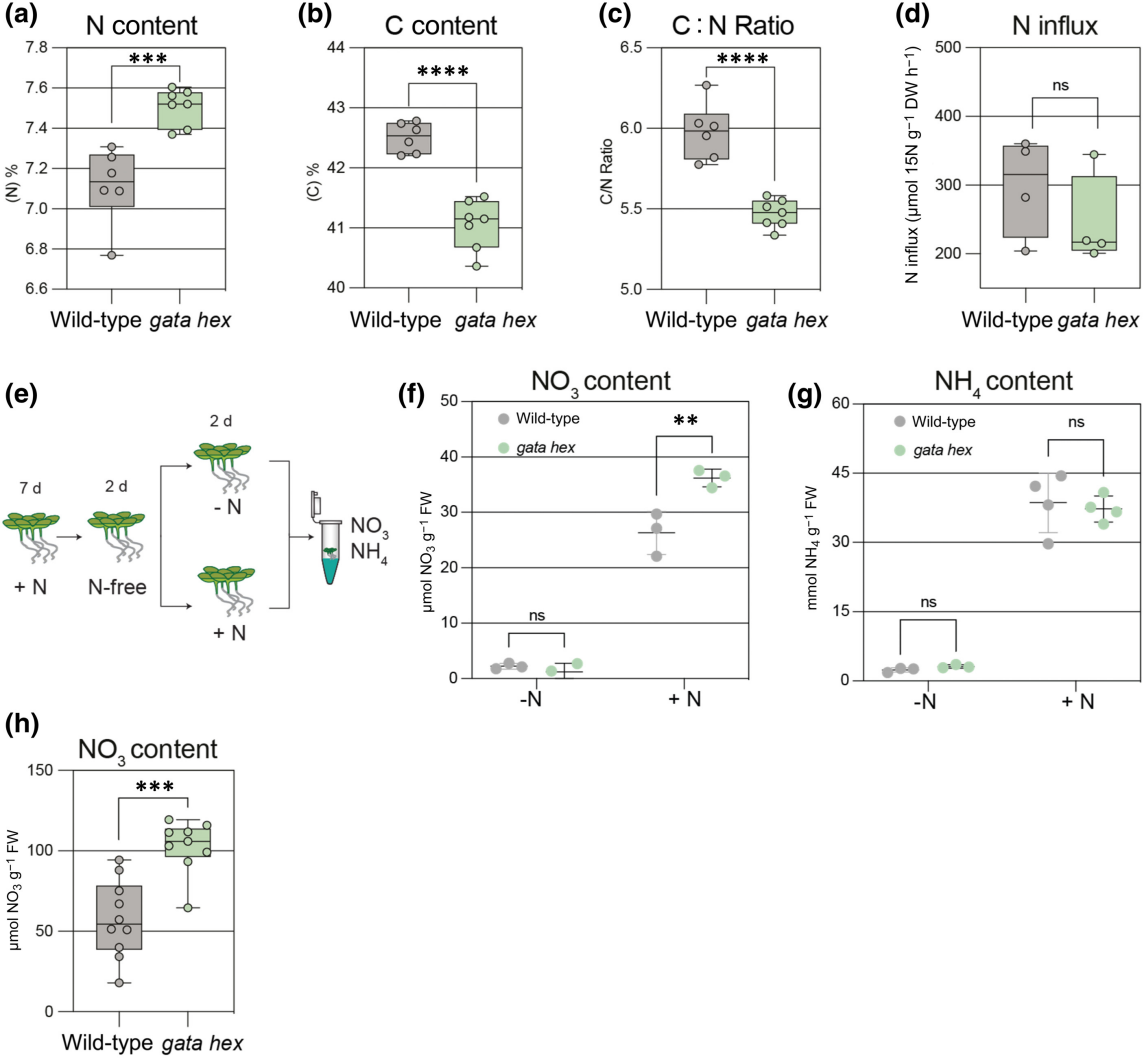
Loss of LLM‐domain B‐GATAs affects endogenous N and C levels as well as NO_3_ uptake in *Arabidopsis thaliana*. (a–c) Total nitrogen (N), carbon (C) content, and C : N ratio in 11‐d‐old *Arabidopsis* wild‐type and *gata* hexuple (gata hex) mutant seedlings. Each data point represents an independent biological replicate (*n* = 9/10 seedlings); the whiskers indicate the full data range (minimum to maximum), boxes show the interquartile range (IQR; spanning the 25^th^–75^th^ percentiles), and the central line indicates the median value of the data distribution. Unpaired *t*‐test; ***, *P* < 0.001; ****, *P* < 0.0001. (d) Nitrate influx in 13‐d‐old seedlings after a 10 min exposure to 10 mM K^15^NO_3_ + 10 mM ^15^NO_3_^15^NH_4_. Each data point represents an independent biological replicate (*n* = 12/15 seedlings); the whiskers indicate the full data range (minimum to maximum), boxes show the interquartile range (IQR; spanning the 25^th^–75^th^ percentiles), and the central line indicates the median value of the data distribution. Unpaired *t*‐test: ns, not significant. (e) Schematic overview of the experimental setup used to determine NO_3_ and NH_4_ as shown in (f) and (g). (f, g) NO_3_^−^ (f) and NH_4_^+^ (g) content in seedlings 2 d after transfer from N‐free (–N) to N‐containing (+N; 10 mM KNO_3_ + 10 mM NO_3_NH_4_) medium, or a corresponding mock treatment (–N; 10 mM KCl). Each data point represents an independent biological replicate (*n* = 10/12 seedlings); bars indicate mean ± SD. Two‐way ANOVA followed by Šídák's multiple comparisons test; **, *P* < 0.01; ns, not significant. (h) Nitrate content in 4‐wk‐old rosettes of the indicated genotypes. Each data point represents an independent biological replicate (*n* = 2 rosettes); the whiskers indicate the full data range (minimum to maximum), boxes show the interquartile range (IQR; spanning the 25^th^–75^th^ percentiles), and the central line indicates the median value of the data distribution. Unpaired *t*‐test: ***, *P* < 0.001.

To examine whether the Arabidopsis *B‐GATA* genes contributed to N metabolism, we quantified the overall N and C content in *Arabidopsis* seedlings following a two‐day period of N starvation after having been grown for 3 d in plates containing 10 mM NH_4_NO_3_ and 10 mM KNO_3_. In this experiment, we found that the *gata hex* mutant exhibited higher N content than the wild‐type, coupled with a reduced carbon content, resulting in a reduced C/N (carbon/nitrogen) ratio (Fig. 9a–c). To examine whether *gata hex* mutants are defective in N uptake, we performed an N influx assay with *gata hex* and the wild‐type (David *et al*., 2019). To this end, we transferred 2‐wk‐old plants to liquid medium lacking N for 2 d before transferring them to a medium containing N (10 mM NH_4_NO_3_ and 10 mM KNO_3_) for 4 h, followed by an additional transfer for a 10‐min treatment to ^15^N‐labeled N (10 mM NH_4_NO_3_ and 10 mM KNO_3_). Isotope analysis following this treatment did, however, not reveal statistically significant changes in N influx under these conditions (Fig. 9d).

We next assayed for the NO_3_^−^ and NH_4_^+^ contents in seedlings transferred for two days to 10 mM NH_4_NO_3_ and 10 mM KNO_3_ following a two‐day N starvation. In this experiment, we measured increased concentrations of NO_3_^−^ in N‐treated *gata hex* seedlings when compared with the wild‐type, while NH_4_ concentrations were comparable between the two genotypes (Fig. 9e–g). Likewise, we measured comparative increases in NO_3_^−^ in the rosettes of 4‐wk‐old *gata hex* plants (Fig. 9h). We concluded that *gata hex* mutants have increased N content and increased NO_3_^−^ levels when compared with the wild‐type, which may not be explained by increased NO_3_^−^ uptake. Since *gata hex* has a decreased N‐responsive growth, these growth differences can thus not be easily explained by decreased N availability in the mutants.

### *Arabidopsis gata hex* mutants are severely impaired in nitrogen‐responsive transcription

**Fig. 10:**
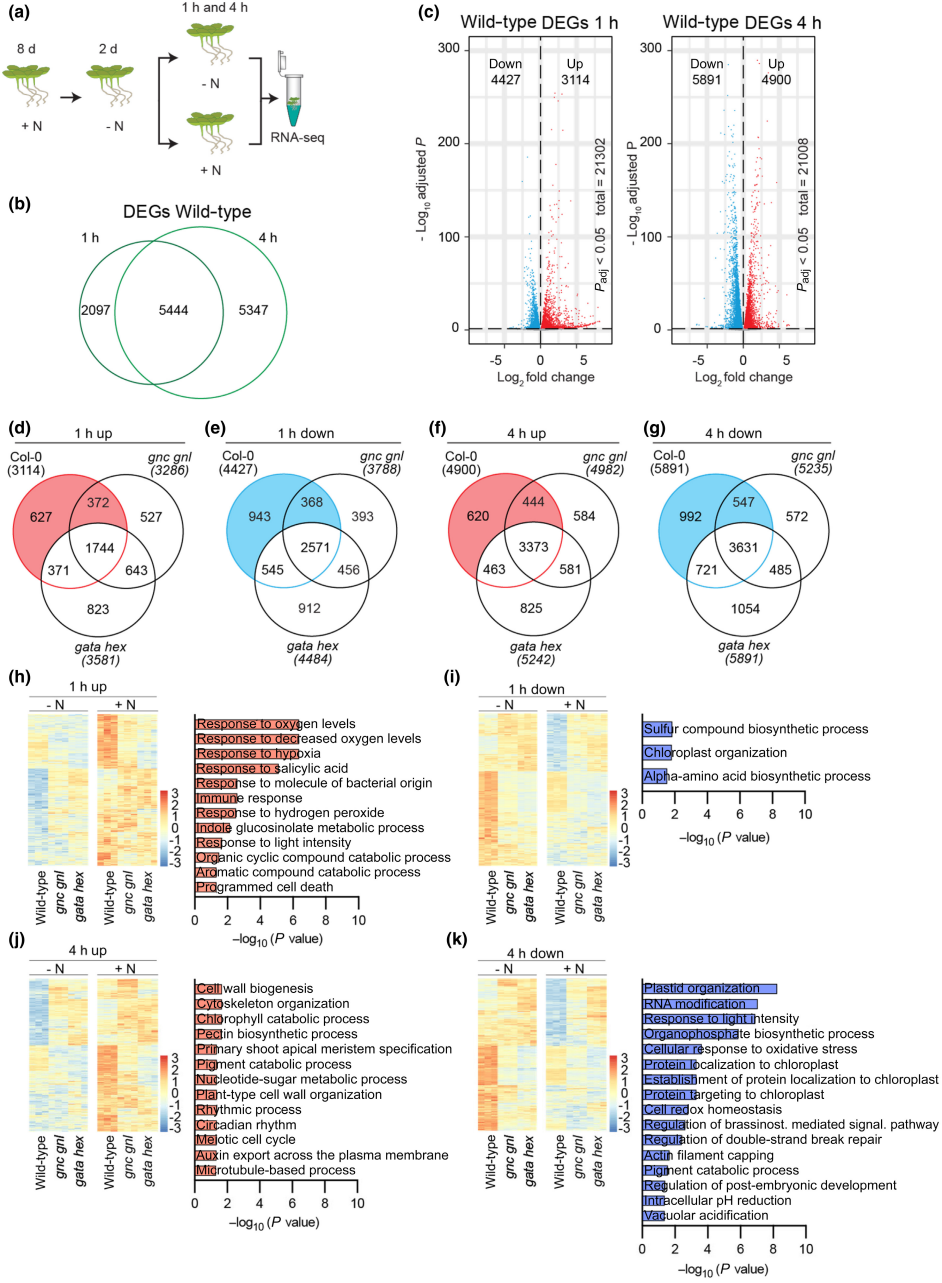
Transcriptomic characterization of the N response in the *Arabidopsis thaliana* wild‐type and the *gnc gnl* and *gata hex* mutants. (a) Schematic overview of the RNA‐seq experiment. Wild‐type and mutant plants were pre‐cultured on N‐containing medium, starved of N for 2 d, and subsequently transferred to either N‐containing (+N; 10 mM KNO_3_ + 10 mM NO_3_NH_4_) or N‐free mock (–N; 10 mM KCl) medium for 1 or 4 h before RNA extraction. (b) Venn diagram showing overlap of differentially expressed genes (DEGs) between +N and –N treatments in *Arabidopsis* wild‐type at the 1‐ h and 4‐h time points. (c) Volcano plots illustrating the distribution of DEGs in the wild‐type at the 1‐ h and 4‐h time points, highlighting genes significantly upregulated or downregulated in response to N. The total number of variables (genes) is provided. (d–g) Venn diagrams comparing DEGs between the wild‐type and the *gnc gnl* and the *gata hex* mutants under +N vs –N conditions, separated into upregulated (d, f) and downregulated (e, g) gene sets at 1 h (d, e) and 4 h (f, g). Color‐coded sections indicate DEGs exclusive to the wild‐type but not in the *gata hex* mutant, which were used for subsequent GO enrichment analysis. (h–k) Heatmaps and corresponding gene ontology (GO) enrichment analyses of DEGs that were regulated in response to N in the wild‐type but not in the *gata hex* mutant, potentially representing AtB‐GATA‐dependent transcripts. Panels show upregulated (h, j) and downregulated (i, k) gene sets at 1 h (h, i) and 4 h (j, k).

To examine gene expression changes in response to N, we exposed 10‐d‐old *gnc gnl*, *gata hex*, and wild‐type seedlings that had been N‐starved for 2 d to N (10 mM NH_4_NO_3_ and 10 mM KNO_3_) (Fig. 10a). A principal component analysis revealed a clear separation of all three replicates of the respective treatments (Fig. S11). After 1 and 4 h of N treatment, we detected 7541 and 10 791 DEGs, respectively, in the wild‐type, which included a comparable number of down‐ and upregulated DEGs at both time points (Fig. 10b,c; Table S8). Gene ontology (GO) analysis showed that N‐regulated DEGs in the wild‐type were enriched for terms such as RNA metabolic process, heat response, photosynthesis, or plastid organization, reflecting a broad response of the plant's physiology to N addition (Fig. S12; Table S9). Among the N‐regulated DEGs in the wild‐type, 627 up‐ and 943 downregulated DEGs from the 1‐h time point, as well as 620 up‐ and 992 downregulated DEGs from the 4‐h time point, were not N‐regulated in *gnc gnl* or *gata hex* (Fig. 10d–g; Table S8c).

Since it can be assumed that the differential regulation of these genes may be the consequence of the loss of the GATA factors, we specifically assessed the functions of the genes that were regulated in the wild‐type but not in *gata hex*. Among the upregulated gene sets, we found that DEGs related to changes in oxygen supply, cell wall biosynthesis, cytoskeleton, and Chl catabolism were regulated in the wild‐type but not in the mutant at the 1‐ or 4‐h time points (Fig. 10h–k; Table S10). Among the downregulated DEGs, genes related to plastid organization and chloroplast function were N‐regulated in the wild‐type but not in the *gata hex* mutant, which we noted with interest since the GNC and GNL GATA factors had previously been implicated not only in Chl biosynthesis but also in chloroplast and amyloplast biology (Chiang *et al*., 2012; Bastakis *et al*., 2018; Sala *et al*., 2023).

We next assessed the regulation of genes that had previously been associated with N biology in *Arabidopsis*, including genes required for N transport, N assimilation, and N‐dependent transcriptional responses (Fig. S13; Table S7a). Among these, several genes related to N transport were differentially regulated in the mutants, as well as genes encoding nitrate reductase (*AtNIA*), nitrite reductase (*AtNIR*), as well as glutamine synthetases (*AtGLU*; *AtGS2*) and asparagine synthetase (*AtASN1*), which showed strong differential regulation when compared with the wild‐type (Fig. S13; Table S7a). The latter expression changes could qualify for the alterations in nitrate levels that we had observed in the mutants after N treatment (Fig. 8). Among the transcriptional regulators, we noted, amongst others, differential regulation of *AtbZIP1*, *AtCDF1*, *AtNLP6*, *AtNLP7*, *as well as AtTGA1 and AtTGA2* (Fig. S13c; Table S7a). When we specifically assessed the regulation of the *B‐GATA* genes in response to the N treatment, we detected an N‐dependent downregulation of *GNC* and *GNL*, but at the same time an upregulation of the B‐GATA factors *GATA15*, *GATA17*, and *GATA17L* (Fig. S13d).

### Strong differential regulation of cytokinin signaling genes in the *gata hex* mutant

Mutants of the *B‐GATA* genes have been associated with reduced Chl biosynthesis (R. Richter *et al*., 2010; Bastakis *et al*., 2018; Schroder *et al*., 2023). When we assessed the RNA‐seq data with regard to the expression of genes required for Chl biosynthesis, we detected the downregulation of a broad set of Chl biosynthesis genes in *gata hex* when compared with the wild‐type, highly similar to previously published gene expression studies related to the role of *B‐GATA* genes in greening (Fig. S14; Table S7).

When we explored genes with a role in cytokinin biosynthesis, we did not note gene expression changes that would qualify to explain the increased cytokinin content detected in the *gata hex* mutant (Fig. S15a–e; Table S7). Strikingly, we found that the expression of the majority of genes with a role in cytokinin signaling was upregulated in the mutant when compared with the wild‐type, suggesting a strong impairment of cytokinin signaling in the mutants, a possible consequence of the elevated cytokinin levels in the mutant or defective feedback regulation (Fig. S15a–e; Table S7).

## Discussion

### *B‐GATA* gene structure

In this study, we characterize the biological function of the four *B‐GATA* genes from the moss *Physcomitrium*. Unlike vascular plant B‐GATAs, but similar to the single B‐GATA in the bryophyte *Marchantia*, moss B‐GATAs contain HAN‐ and LLM‐domains (Schwechheimer *et al*., 2022). The biochemical activity of either domain is unknown, and mutation analyses of *B‐GATA* genes in vascular plants showed that both domains are individually required for full function of the respective B‐GATA subtypes (Whipple *et al*., 2010; Behringer *et al*., 2014). Given the co‐occurrence of both domains in bryophyte B‐GATAs, it had previously been proposed that the common land plant B‐GATA ancestor may have contained both domains (Schwechheimer *et al*., 2022). It has also been suggested that the two domains are functionally interdependent and that, consequently, heterodimerization between HAN‐ and LLM‐domain B‐GATAs may be required for full B‐GATA protein activity in vascular plants (Zhang *et al*., 2013; Schwechheimer *et al*., 2022). However, the absence of phenotypes typically observed in mutants of *HAN‐domain B‐GATAs*, for example abnormal flower morphology and embryo lethality, in higher‐order mutants of *LLM‐domain B‐GATAs*, as in the *gata hex* mutant used in this study, which carries mutations in all six *Arabidopsis LLM‐domain B‐GATA* genes and was previously unavailable, now argues against such functional interdependence between the two B‐GATA subfamilies (Zhao *et al*., 2004; Zhang *et al*., 2013; Schwechheimer *et al*., 2022; Schroder *et al*., 2023).

The four LLM‐domain B‐GATAs from *Physcomitrium* exhibit very high sequence conservation and are essentially identical in the conserved zinc finger domain, the HAN‐domain, and the LLM‐domain (Fig. 1). In vascular plants, the zinc finger DNA‐binding domain among the B‐GATAs is highly conserved, but its sequence allows predicting whether a B‐GATA factor contains a HAN‐ or an LLM‐domain (Behringer *et al*., 2014; Schwechheimer *et al*., 2022). The presence of a basic lysine (K) residue in the zinc finger domain of the four *Physcomitrium* B‐GATAs, as well as in the single *Marchantia* B‐GATA, at a position where vascular plant HAN‐domain B‐GATAs have a serine (S), suggests that bryophyte B‐GATAs, from this perspective, are more closely related to LLM‐domain B‐GATAs than to HAN‐domain B‐GATAs of vascular plants (Fig. 1).

### Functional redundancy among the *PpB‐GATAs*

Although we lack functional evidence from dedicated genetic studies, we assume that the four B‐GATAs are functionally redundant. Expression analyses using publicly available data indicate that their expression has diverged across different cell and tissue types. We generated single, double, and triple mutants with defects in *PpB‐GATA1*, *PpB‐GATA3*, and *PpB‐GATA4*. We unsuccessfully used two alternative approaches to obtain a mutant for the fourth family member, *PpB‐GATA2*. Whether *PpB‐GATA2* harbors a critical biological role that would preclude mutant survival is difficult to assess. The relatively low expression of the gene, along with the presumed functional redundancy among the *PpB‐GATAs*, would be an argument against such an essential role.

### *Ppb‐gata1/3/4* mutants have reduced Chl and carotinoid levels

In order to be able to perform the analysis with a reasonable effort and, in part, cost, we have focused our analysis primarily on the study of the moss *Ppb‐gata1/3/4* triple mutant. We found that the *Ppb‐gata1/3/4* triple mutant has reduced Chl and carotenoid contents, and thereby the moss mutant phenotypically resembles *B‐GATA* gene mutants from *Arabidopsis* and *Marchantia* (Ranftl *et al*., 2016; Bastakis *et al*., 2018; Schroder *et al*., 2023) (Bi *et al*., 2005; Hudson *et al*., 2011). The reduced expression of several Chl biosynthesis genes in *Ppb‐gata1/3/4*, notably the downregulation of multiple *HEME* gene orthologs, *CHLI*, and *PORA*, as observed in our RNA‐seq analysis, may account for the observed Chl reduction. As a note of caution, we would like to emphasize here the fact that the Chl quantifications, as well as the cytokinin quantifications discussed below, were obtained from plants grown on N‐containing medium, whereas the RNA‐seq data refer to plant material from N‐starved plants.

### N‐responsive growth is defective in *B‐GATA* gene mutants

Importantly, we found that *Ppb‐gata1/3/4* mutants are defective in the formation of protonemata typically observed in moss in growth medium containing the N sources NO_3_ and NH_4_. Similarly, we observed reduced growth in response to N supply when growing *Arabidopsis gnc gnl* double mutants and even more so when growing *gata hex* hexuple mutants on soil containing increased N contents or supplemented with fertilizer. Further, we observed reduced systemic growth in *gata hex* when roots were grown on N‐depleted medium in split‐root assays.

### Transcriptional responses to N strongly depend on *B‐GATA* genes

These observations triggered our interest in studying the role of the B‐GATAs in their transcriptional response to N after N‐starvation, initially in *Physcomitrium* and subsequently in *Arabidopsis*. In both species, we found a substantial reduction (*Physcomitrium* 42%; *Arabidopsis* 27%) in the number of genes regulated by N‐supply in the wild‐type after 1 and 4 h, suggesting that the *B‐GATA* genes make a significant contribution to the transcriptional N response.

Functional characterization of the classes of genes required for N uptake, assimilation, and response revealed differential regulation of genes belonging to all functional classes in the moss and *Arabidopsis* mutants. In combination with physiological analysis, we could conclude, however, that neither mutant was defective in N uptake. Similarly, the expression of genes required for N assimilation, specifically the enhanced N‐induced expression of genes encoding for nitrate reductases (moss *Pp6c10_5150*, *Pp6c10_5180* and *Arabidopsis AT1G77760*, *AT1G37130*) and nitrite reductases (*Pp6c25_1290*, *Pp6c16_12770*, *Pp6c1_8170* and *AT2G15620*), as well as for the genes encoding for glutamine (*Pp6c2_2380*, *Pp6c20_11610* and *AT3G60120*) and asparagin synthetases (*AT3G47340 and AT5G65010*), argues for enhanced N assimilation in the mutants. In line with these observations, our physiological analyses found increased NO_3_ contents in mutants after N supplementation of N‐starved plants, at least in *Arabidopsis*, whereas NO_3_ contents in moss were below the detection limit. In moss, in turn, NH_4_ contents were reduced when N‐starved *Ppb‐gata1/3/4* mutants were supplemented with NH_4_. From this analysis, we can conclude that the transcriptional responses for N assimilation genes are strongly affected in the *B‐GATA* gene mutants from both species, which may correlate with altered N metabolism in the respective plants.

Transcriptional responses downstream from N supply have been studied extensively, and time‐resolved kinetic analyses have identified transcription factors that respond to N supply. Several of the relevant studies identify *GATA* motifs as overrepresented promoter elements in N‐regulated genes, arguing from this angle for an important role of GATA factors as regulators of N‐responsive growth. Many of these transcription factors are differentially regulated in the moss *Ppb‐gata1/3/4*, as well as in the *Arabidopsis gnc gnl*, and *gata hex* mutants, for example *CDF1* (*Pp6c15_10030* and *AT5G23040*), *NLP6* (*Pp6c19_1500* and *AT1G64530*), *NLP7* (*Pp6c17_1870* and *AT4G24020*), *TCP20* (*Pp6c10_10790* and *AT3G27010*), *TGA1* (*Pp6c3_4570* and *AT5G65210*), TGA4 (*Pp6c14_2590* and *AT5G10030*), which invites the conclusion that at least some of the transcriptional responses downstream from N are conserved between moss and *Arabidopsis* (Ristova *et al*., 2016; Ruffel *et al*., 2016; Varala *et al*., 2018; Brooks *et al*., 2019).

### B‐GATA gene functions can be linked to cytokinin biology

Intriguingly, the growth defect of the *Physcomitrium Ppb‐gata1/3/4* mutant resembles that of moss lines with impaired cytokinin‐regulated development caused by overexpression of the KNOTTED family transcription factor *MKN2* or by misexpression of an *ISOPENTENYL TRANSFERASE* gene in the *mkn2* mutant (Coudert *et al*., 2019). In *Arabidopsis*, *GNL* (*GNC‐LIKE*) was previously identified as a strongly cytokinin‐responsive gene, which led to its alternative name *CGA1* (*CYTOKININ‐RESPONSIVE GATA1*). We have previously shown that the transcription of all six *Arabidopsis LLM‐domain B‐GATA* genes can be induced by cytokinin, albeit less strongly than that of *GNL/CGA1* (Naito *et al*., 2007; Ranftl *et al*., 2016). In the same study, we found that a mutant lacking five of the six *Arabidopsis* LLM‐domain B‐GATAs exhibits defects in phyllotaxy, floral organ initiation, accessory meristem formation, and senescence, phenotypes attributable to impaired cytokinin signaling (Ranftl *et al*., 2016). Similarly, *Mpb‐gata1* mutants from the liverwort *Marchantia* display gemma cup formation defects that phenocopy those of mutants in the MYB transcription factor *GCAM1* (*GEMMA CUP‐ASSOCIATED MYB1*) (Schroder *et al*., 2023). *GCAM1* is a master regulator of vegetative reproduction in *Marchantia* and has recently been linked to the cytokinin pathway in this species (Komatsu *et al*., 2025). Here, we show that moss *Ppb‐gata1/3/4* and *Arabidopsis gata hex* mutants accumulate higher cytokinin levels than their respective wild‐type counterparts. In mutants from both species, numerous genes associated with cytokinin biosynthesis (*Physcomitrium*) or signal transduction (*Arabidopsis*) are differentially expressed, indicating that cytokinin‐responsive transcription or feedback regulation in the cytokinin pathway are misregulated. The reduced accumulation of Chl, as well as the reduced transcription of Chl biosynthesis genes and other aspects of chloroplast biology, may, at least in *Arabidopsis*, also be explained by a reduction of cytokinin signaling (Chiang *et al*., 2012; Ranftl *et al*., 2016). Collectively, these findings suggest that B‐GATAs contribute to cytokinin‐regulated growth in mosses, bryophytes, and angiosperms, pointing to an evolutionary conservation of this role for B‐GATA factors in land plants.

In summary, our study identifies an important role for the *B‐GATA* genes in mediating N‐dependent growth in *Physcomitrium* patens and *Arabidopsis thaliana*. The increases in cytokinin levels in mutants from both species and the strongly impaired transcriptional responses, in combination with the impairment of N‐responsive growth, suggest that the *B‐GATA* genes may translate the role that has been attributed to cytokinin as a systemic regulator of N‐mediated plant growth. Through our studies, we provide solid and strong proof for the anticipated but as yet unproven role of plant GATAs in N‐dependent plant growth regulation. Dedicated studies are needed to examine the functional and potentially direct relationship between the GATA factors and the reported N‐dependent gene expression changes.

## Competing interests

None declared.

## Author contributions

DZ and CS conceived all biological experiments. PMS generated the *Physcomitrium Ppb‐*gata gene mutants. IP and ON performed and analyzed the cytokinin quantifications. XD and KS analyzed the *Ppb‐gata* mutant genome sequences. RS supported and analyzed the ^15^N uptake studies.

## Disclaimer

The New Phytologist Foundation remains neutral with regard to jurisdictional claims in maps and in any institutional affiliations.

## Supporting information


**Fig. S1** Phylogenetic tree of B‐GATA proteins from *Physcomitrium patens* (Pp), *Marchantia polymorpha* (Mp), and *Arabidopsis thaliana* (At).
**Fig. S2** *PpB‐GATA* gene expression levels across different tissues in *Physcomitrium patens*.
**Fig. S3** Genotypes of *Physcomitrium patens Ppb‐gata* mutant alleles obtained by CRISPR/Cas9‐based mutagenesis.
**Fig. S4** *PpB‐GATA1* overexpression suppresses growth and enhances Chl and carotenoid accumulation in *Physcomitrium patens*.
**Fig. S5** Principal component analysis (PCA) of transcriptomic profiles from *Physcomitrium patens* Reute and the *Ppb‐gata1/3/4* triple mutant.
**Fig. S6** Gene ontology (GO) enrichment analysis of differentially expressed genes (DEGs) in response to nitrogen in *Physcomitrium patens* Reute.
**Fig. S7** Differential regulation of nitrogen transport, metabolism, and transcription regulation genes may underlie the reduced N response in *Physcomitrium patens Ppb‐gata1/3/4* mutants.
**Fig. S8** Altered expression of Chl biosynthesis genes may contribute to reduced Chl accumulation in *Physcomitrium patens Ppb‐gata1/3/4* mutants.
**Fig. S9** Expression profiles of cytokinin biosynthesis genes in *Physcomitrium patens Ppb‐gata1/3/4* mutants.
**Fig. S10** Loss of *GNC* and *GNL* leads to a partial suppression of N‐dependent growth responses in *Arabidopsis thaliana*.
**Fig. S11** Principal component analysis (PCA) of transcriptomic profiles from *Arabidopsis thaliana* and *b‐gata* mutants.
**Fig. S12** Gene ontology (GO) enrichment analysis of differentially expressed genes (DEGs) in response to N in *Arabidopsis thaliana*.
**Fig. S13** Differential regulation of nitrogen transport, metabolism, and transcription regulation genes may underlie the reduced N response in *gnc gnl* and *gata hex* mutants from *Arabidopsis thaliana*.
**Fig. S14** Altered expression of Chl biosynthesis genes in *Arabidopsis thaliana gnc gnl* and *gata hex* mutants.
**Fig. S15** Expression profiles of cytokinin biosynthesis genes in *Arabidopsis thaliana gnc gnl* and *gata hex* mutants.
**Methods S1** Supplemental materials and methods.

**Table S1** List of primers used in the study.

**Table S2** Results of the whole genome sequencing of *Physcomitrium patens Ppb‐gata* mutant lines.

**Table S3** Results of the cytokinin quantifications in *Physcomitrium patens* and *Arabidopsis thaliana* wild‐type and *gata* mutants.

**Table S4** Results of the RNAseq analysis conducted in *Physcomitrium patens*.

**Table S5** Results from a gene ontology (GO) enrichment analysis results from g:Profiler of differentially expressed genes (DEGs) in nitrogen (10 mM KNO_3_ + 5 mM (NH_4_)_2_C_4_H_4_O_6_) vs mock (10 mM KCl) conditions in *Physcomitrium patens* Reute.

**Table S6** Results from a gene ontology (GO) enrichment analysis results from g:Profiler of differentially expressed genes (DEGs) in nitrogen (10 mM KNO_3_ + 5 mM (NH_4_)_2_C_4_H_4_O_6_) vs mock (10 mM KCl) conditions in *Physcomitrium patens* Reute but not in *Ppb‐gata1/3/4*, grouped according to up‐ or downregulation and time point as shown in Fig. 6.

**Table S7** Lists of genes from *Arabidopsis thaliana* and their orthologues from *Physcomitrium patens* involved in nitrogen response, Chl biosynthesis, and cytokinin biosynthesis and response, as analyzed for this study.

**Table S8** Results of the RNAseq analysis conducted in *Arabidopsis thaliana*.

**Table S9** Results from a gene ontology (GO) enrichment analysis results from ClusterProfiler of differentially expressed genes (DEGs) in nitrogen (10 mM KNO_3_ + 10 mM NH_4_NO_3_) vs mock (10 mM KCl) conditions in *Arabidopsis thaliana* (Col‐0) wild‐type, grouped according to up‐ or downregulation and time point as shown in Fig. S12.

**Table S10** Results from a gene ontology (GO) enrichment analysis from ClusterProfiler of differentially expressed genes (DEGs) in nitrogen (10 mM KNO_3_ + 10 mM NH_4_NO_3_) vs mock (10 mM KCl) conditions in *Arabidopsis thaliana* (Col‐0) that are not nitrogen‐responsive in the *gata hex* mutant, grouped according to up‐ or downregulation and time point as shown in Fig. 10h–k.
Please note: Wiley is not responsible for the content or functionality of any Supporting Information supplied by the authors. Any queries (other than missing material) should be directed to the *New Phytologist* Central Office.

## Data Availability

Raw data of both experiments were deposited in the repository of NFDI4plants under Madland ARC Schwechheimer (http://git.nfdi4plants.org) and can be retrieved at the following link (Weil *et al*., 2023) doi: 10.60534/66p52‐bct41.
