# Supplementary material for: B‐GATA factors are required for nitrogen‐responsive growth in Physcomitrium patens and Arabidopsis thaliana
Source: New Phytol. 2026 Jan 9;249(6):2919–37. doi: 10.1111/nph.70887 (PMC12917456; doi:10.1111/nph.70887)
Supplement: Supplementary file 1 — Fig. S1 Phylogenetic tree of B‐GATA proteins from Physcomitrium patens (Pp), Marchantia polymorpha (Mp), and Arabidopsis thaliana (At). Fig. S2 PpB‐GATA gene expression levels across different tissues in Physcomitrium patens. Fig. S3 Genotypes of Physcomitrium patens Ppb‐gata mutant alleles obtained by CRISPR/Cas9‐based mutagenesis. Fig. S4 PpB‐GATA1 overexpression suppresses growth and enhances Chl and carotenoid accumulation in Physcomitrium patens. Fig. S5 Principal component analysis (PCA) of transcriptomic profiles from Physcomitrium patens Reute and the Ppb‐gata1/3/4 triple mutant. Fig. S6 Gene ontology (GO) enrichment analysis of differentially expressed genes (DEGs) in response to nitrogen in Physcomitrium patens Reute. Fig. S7 Differential regulation of nitrogen transport, metabolism, and transcription regulation genes may underlie the reduced N response in Physcomitrium patens Ppb‐gata1/3/4 mutants. Fig. S8 Altered expression of Chl biosynthesis genes may contribute to reduced Chl accumulation in Physcomitrium patens Ppb‐gata1/3/4 mutants. Fig. S9 Expression profiles of cytokinin biosynthesis genes in Physcomitrium patens Ppb‐gata1/3/4 mutants. Fig. S10 Loss of GNC and GNL leads to a partial suppression of N‐dependent growth responses in Arabidopsis thaliana. Fig. S11 Principal component analysis (PCA) of transcriptomic profiles from Arabidopsis thaliana and b‐gata mutants. Fig. S12 Gene ontology (GO) enrichment analysis of differentially expressed genes (DEGs) in response to N in Arabidopsis thaliana. Fig. S13 Differential regulation of nitrogen transport, metabolism, and transcription regulation genes may underlie the reduced N response in gnc gnl and gata hex mutants from Arabidopsis thaliana. Fig. S14 Altered expression of Chl biosynthesis genes in Arabidopsis thaliana gnc gnl and gata hex mutants. Fig. S15 Expression profiles of cytokinin biosynthesis genes in Arabidopsis thaliana gnc gnl and gata hex mutants. Methods S1 Supplemental materials and met [file NPH-249-2919-s011.pdf]

New Phytologist Supporting Information

Article title: B-GATA factors are required for nitrogen-responsive growth in *Physcomitrium patens* and *Arabidopsis thaliana*

Authors: Dario Zappone, Peter Michael Schröder, Ivan Petřík, Xiao Dong, Rudi Schäufele, Korbinian Schneeberger, Ondřej Novák, Claus Schwechheimer

Article acceptance date: 8 December 2025

METHODS S1

Sequence alignments and phylogeny construction. To generate a phylogenetic tree of the B-GATAs, B-GATA protein sequences were retrieved from Phytozome (*Physcomitrium patens (Hedw.) Mitt.*, *Marchantia polymorpha L.*) and TAIR (*Arabidopsis thaliana (L.) Heynh.*). Protein sequences were initially aligned using full-length sequences and subsequently realigned, focusing on the zinc finger domains using Geneious v11.1.2 (https://www.geneious.com). Phylogenetic trees were constructed using the Jukes-Cantor genetic distance model and the Neighbor-Joining method implemented in Geneious v11.1.2.

CRISPR/Cas9-mediated mutagenesis. To examine the effects of the loss of *PpB-GATA* gene function, *Ppb-gata* mutants were generated in *Physcomitrium patens* Reute by CRISPR/Cas9-mediated mutagenesis. *PpB-GATA1 (Pp6c22_5780/Pp3c22_11040), PpB-GATA2 (Pp6c21_8280/Pp3c21_15110), PpB-GATA3 (Pp6c18_6710/Pp3c18_14800), and PpB-GATA4 (Pp6c22_5810/ Pp3c22_11080)* genes were targeted with one gRNA each (Supplemental Table S1). For CRISPR/Cas9-mediated mutagenesis, a fragment containing attB sites, the *snRNA U6* promoter, and the chemically synthesized gRNAs (GeneArt, ThermoFisher Scientific, Regensburg, Germany) was cloned into pDONR207 (Invitrogen, Life Technologies, Carlsbad, CA) (Collonnier et al., 2017). For transformation, *Physcomitrium* protoplasts were extracted and transformed with combinations of pDONR207 constructs targeting the four *B-GATA* genes as previously described (Ashton et al., 1979). After transformation, plants were regenerated for one week from the transformed protoplasts on cellophane disks positioned on BCDAT medium supplemented with 0.33 M mannitol (Ashton et al., 1979). Selection of regenerated protoplasts was carried out by transferring the cellophane disks onto new medium containing 50 µg/mL G418 (Duchefa, Harlem, The Netherlands). Genomic DNA was

extracted from resistant plants using the CTAB (cetyltrimethylammonium-bromide) method and genotyped by DNA sequencing following PCR amplification of the gene region of interest (Supplemental Table S1) (Moody et al., 2021). Mutant alleles were classified based on sequence insertions and deletions in the gene region of interest.

To obtain a mutant of *PpB-GATA2* by homologous recombination, the 5'- and 3'-sequences of the untranslated regions of the *PpB-GATA2* gene were amplified using nested PCR to ensure target specificity. The hygromycin resistance cassette was inserted between the two homologous recombination arms in the Golden Gate destination vector pGGZ003 and subsequently used for protoplast transformation as described above (Supplemental Table S1). No mutants of *PpB-GATA2* were obtained from this procedure, which is listed here only for completeness.

Genomic DNA sequencing of *Physcomitrium Ppb-gata* mutants. To obtain high-quality genomic DNA from *Physcomitrium Ppb-gata* mutants for genomic sequencing, DNA was extracted from 50 mg of plant material, pooling together material from three different colonies of four-week-old *Physcomitrium Ppb-gata* mutants, using the DNeasy® Plant Pro Kit (Qiagen, Hilden, Germany) and quality-checked using the High Sensitivity DNA assay on a Fragment Analyzer 5300 System (Agilent Technologies, Santa Cruz, CA). Genomic DNA libraries were prepared using the NEBNext Ultra II DNA Kit (New England Biolabs). Library concentrations were determined using the Qubit® dsDNA HS Assay Kit (ThermoFisher Scientific, Waltham, MA) and additionally quantified via qPCR with the NEBNext® Library Quant Kit (New England Biolabs) on a ViiA7™ Real-Time PCR System (ThermoFisher Scientific, Waltham, MA). After dilution to 1.85 nM and denaturation with NaOH to ensure the presence of single-stranded DNA fragments, libraries were sequenced on an Illumina NovaSeq® 6000 300-cycle flow cell (400M Reads) using the Illumina NovaSeq® XP 2-Lane Kit (Illumina).

To identify polymorphisms in the *Physcomitrium* mutants, a combination of short-read variant calling and sample-specific k-mer analysis was used. Illumina reads were adapter-trimmed using Skewer v0.2.1 (Jiang et al., 2014), then aligned to the *Physcomitrium* v3.3 genome (Phytozome Genome ID: 318) with BWA-MEM v0.7.17-r1188 (Li, 2013). Aligned reads were sorted using Samtools v1.21 (Danecek et al., 2021) and PCR duplicates were marked with Picard (http://broadinstitute.github.io/picard). Variant calling was performed using GATK

HaplotypeCaller v4.2.4.1 in haploid mode (Van der Auwera, 2017). SNPs and INDELs were selected and filtered based on quality metrics (e.g., QD < 2.0, FS > 60.0, MQ < 40.0). Filtered variants were then used for base quality score recalibration (BQSR), followed by a second round of GATK variant calling using the recalibrated reads. Joint genotyping across samples was performed via CombineGVCFs and GenotypeGVCFs.

To reduce false positives caused by sequencing or alignment errors, we further filtered variants using sample-specific unique 31-mers. Meryl v1.4 (Rhie et al., 2020) was used to count and subtract k-mers across samples to isolate unique k-mers for each mutant. Reads containing these k-mers were extracted with meryl-lookup, realigned to the reference, and regions with ≥10x coverage were merged using bedtools v2.31.1 (Quinlan and Hall, 2010). GATK-called variants were intersected with these unique k-mer-supported regions to retain only high-confidence mutations.

Validated variants were manually inspected in IGV v2.13.0 (Robinson et al., 2011) for confirmation and then functionally annotated using SnpEff v5.1d (Cingolani et al., 2012) with a custom database built from the reference genome and annotation.

Confocal microscopy of protonemata. Confocal microscopy and analysis of *Physcomitrium* protonemata were performed on two-week-old tissue grown on 10 mM KNO_3-only medium, sandwiched between two layers of cellophane, to promote two-dimensional growth of the protonemata cells suitable for imaging. Cells were stained with a drop of Calcofluor White Stain (CSW) and 10% KOH prior to imaging using an Olympus FV1000 Laser Scanning Confocal Microscope with a 10x objective lens. Laser excitation was set to 405 nm. Confocal images were exported using Fiji (Image J), and quantification of cell division plane angles was performed as previously described (Coudert et al., 2019). Statistical analysis and graphic display were performed with the help of GraphPad Prism v. 10.

***PpB-GATA1* overexpression lines and molecular cloning.** To generate the *Physcomitrium PpB-GATA1* overexpression lines, the coding sequence of *PpB-GATA1* (*Pp6c22_5780*) was tagged at the C-terminus with an mCitrine tag and placed under the control of the constitutive *PpActin5* promoter (*Pp6c10_9120*) in pGGX000 (Lampropoulos et al., 2013). The kanamycin resistance cassette + 3' HR *PpARPC2* was assembled in one entry vector (pGGF000) by performing an overlap PCR.

Physcomitrium protoplast transformation and selection were performed as described above (Ashton et al., 1979). Homologous recombination was performed targeting *PpARPC2 (*Pp6c1_16850), a redundant gene in *Physcomitrium*, as described (https://sites.dartmouth.edu/bezanillalab/moss-methods/overexpression/). Regenerated protoplasts were identified by growth on media containing 50 µg/mL kanamycin. Genotyping was performed by PCR, RT-qPCR, and DNA sequencing (Supplemental Table S1).

Reverse transcription-quantitative PCR (RT-qPCR). For RT-qPCR, RNA extraction was performed from 50-100 mg of *Physcomitrium* material, using the NucleoSpin RNA kit (Macherey-Nagel, Düren, Germany). cDNA was generated using oligo(dT) primers and RevertAid reverse transcriptase (ThermoFisher Scientific, Waltham, MA) according to the manufacturer's instructions. Takyon No ROX SYBR MasterMix (Eurogentec, Liege, Belgium) was used for the qRT-PCR in a CFX384 Real-Time System Cycler (BioRad, Feldkirchen, Germany) with the following protocol: step 1, 95 °C for 3:00 min; step 2, 95 °C for 0:10 min; step 3, 55 °C for 0:30 min followed by 39 repeats of steps 2 and 3 using gene-specific primers (Supplemental Table S1). Relative quantification was calculated according to the ΔC_t method using the housekeeping genes *PpSTP2A (Pp6c25_1380*) and *PpADE* (*Pp6c8_8410*) (Livak and Schmittgen, 2001; Le Bail et al., 2013).

***Physcomitrium* and *Arabidopsis* growth conditions.** Unless otherwise stated, *Physcomitrium* single gametophores were cultivated on BCDAT medium supplemented with 10 mM KNO_3 and 5 mM $(NH_4)_2C_4H_4O_6$ as N sources and solidified with 0.8% plant agar for asexual propagation and phenotyping (http://moss.nibb.ac.jp/protocol.html). Growth on individual N sources was achieved by supplying either 10 mM KNO_3 or 5 mM $(NH_4)_2C_4H_4O_6$ alone. Cultures were maintained under 80 µmol m^{-2} s^{-1} white light (L58W/830, Lumilux warm white, 5200 lm; Osram, Munich, Germany) at 22 °C in long-day conditions (16 h light/8 h dark) using MLR-351 plant growth chambers (Sanyo Denki, Eschborn, Germany).

For *in vitro* growth of *Arabidopsis*, unless otherwise stated, seeds were stratified for 2–3 days at 4 °C in the dark on ½ strength Murashige & Skoog (MS) modified medium without N (Biotrend, Köln, Germany) to control the N sources and concentrations. Plants were then grown under 80 µmol m^{-2} s^{-1} white light (L58W/830, Lumilux warm

white, 5200 lm, Osram, Munich, Germany) at 22 °C in long-day conditions (16 h light/8 h dark) in MLR-351 plant growth chambers (Sanyo Denki, Eschborn, Germany).

For soil-based growth experiments of *Arabidopsis* on differential soil/sand mixtures, seeds were stratified at 4 °C in the dark for 3 days, then germinated on standard soil (Floragard Floraton 3, Floragard Vertriebs-GmbH, Oldenburg, Germany) under white light (120 µmol m^{-2} s^{-1}) at 21 °C for 1 week. Subsequently, seedlings were transferred to different soil/sand mixtures and cultivated for an additional 3 weeks under the same light and temperature conditions (120 µmol m^{-2} s^{-1}, 21 °C) in long-day cycles (16 h light/8 h dark) in MLR-351 plant growth chambers (Sanyo Denki, Eschborn, Germany).

For fertilizer treatment of *Arabidopsis*, Complete Pflanzendünger (COMPO, Münster, Germany) was applied once weekly according to the manufacturer's instructions using 7 mL diluted in 2 L water for a maximum final N concentration of (approximately) 12 mM NO_3 and 4.3 mM NH_4. Plant growth was assessed by measuring rosette fresh weight, and plant material was frozen for subsequent NO_3 and NH_4 quantification.

***Physcomitrium* and *Arabidopsis* imaging and area quantification.** The area covered by *Physcomitrium* colonies and *Arabidopsis* rosettes was quantified from digital photographs taken with a Sony α6100 camera (Sony, Berlin, Germany). Plant areas were determined by selecting the characteristic green color, outlining the regions of interest, and measuring them in Adobe Photoshop (Adobe Inc., San José, CA), with manual adjustments where needed. Circularity from *Physcomitrium* plants was calculated using the formula: circularity = 4π × area/perimeter^2.

***Physcomitrium* chlorophyll and carotenoid quantifications.** To extract and quantify chlorophyll and carotenoids from *Physcomitrium*, 10–40 mg fresh weight of four-week-old plants grown under long-day conditions (16 h light/8 h dark) on BCDAT medium were harvested, flash-frozen in liquid N_2, and homogenized with steel beads using a TissueLyser II (Qiagen, Hilden, Germany). The homogenate was extracted in 80% (v/v) aqueous acetone on ice in the dark, following the method described by Porra (1989). After centrifugation at 16,000 × *g* for 15 min at 4 °C, the absorbance of the supernatant was measured at 470 nm, 645 nm, 663 nm, and 720 nm using a Tristar 5 plate reader (Berthold Technologies, Bad Wildbad, Germany). Chlorophyll and carotenoid concentrations were calculated using previously established equations,

with correction for background absorbance at 720 nm (Lichtenthaler, 1987; Porra, 1989).

$$Chl_a\left(\frac{\mu g}{mg\ FW}\right) = [12.25 \times (A^{663nm} - A^{720nm}) - 2.55\ \times (A^{645nm} - A^{720nm})]\ \times \frac{V}{W}$$

$$Chl_b\left(\frac{\mu g}{mg\ FW}\right) = [20.31 \times (A^{645nm} - A^{720nm}) - 4.91\ \times (A^{663nm} - A^{720nm})]\ \times \frac{V}{W}$$

$$Chl_{a+b}\left(\frac{\mu g}{mg\ FW}\right) = [17.76 \times (A^{645nm} - A^{720nm}) + 7.34\ \times (A^{663nm} - A^{720nm})]\ \times \frac{V}{W}$$

$$Car_{x+c}\left(\frac{\mu g}{mg\ FW}\right) = \left[\frac{1000 \times (A^{470nm} - A^{720nm}) - 1.82\ \times Chl_a - 85.02\ \times Chl_b}{198}\right] \times \frac{V}{W}$$

Cytokinin quantifications from *Physcomitrium* and *Arabidopsis*. To quantify cytokinin concentrations, four-week-old *Physcomitrium* colonies grown on BCDAT or four-week-old *Arabidopsis* rosettes from plants grown on normal soil were flash-frozen in liquid N_2. To ensure homogeneity of samples, 3-5 *Physcomitrium* colonies or 2-3 *Arabidopsis* rosettes were pooled as one biological replicate. The frozen plant material was first pulverized with metal beads and, subsequently, 10 mg (+/- 1) of fresh weight for each biological replicate was weighed and used for cytokinin determination.

Cytokinin metabolites were determined from a minimum of six biological replicates according to a modified protocol of a previously published method (Simura et al., 2018). Briefly, samples containing 10 mg fresh weight of biological material were extracted in an aqueous solution of 50% (v/v) acetonitrile. A mixture of stable isotope-labelled standards of phytohormones was added to validate the LC-MS/MS method. Crude extracts were loaded onto conditioned Oasis HLB columns (30 mg/1 ml, Waters, Milford, MA, USA) and washed with an aqueous solution of 30% (v/v) acetonitrile. Flow-through fractions containing purified analytes were collected and evaporated to dryness in a vacuum evaporator. The chromatographic separation was performed using an Acquity I class system (Waters, Milford, MA, USA) equipped with an Acquity UPLC® CSH C18 RP column (150 × 2.1 mm, 1.7 µm; Waters, Milford, MA, USA). Effluent was analyzed using a triple quadrupole mass spectrometer (Xevo™ TQ-XS, Waters, Milford, MA, USA) equipped with an electrospray ionization source. Data were processed with Target Lynx V4.2 software, and final concentration levels

of phytohormones were calculated using isotope dilution (Rittenberg and Foster, 1940).

Analysis of *Arabidopsis* root growth in split-root assays. A previously published protocol was adapted for the split-root assay with *Arabidopsis* plants (Ruffel et al., 2011). *Arabidopsis* seeds were stratified for 2–3 days at 4 °C in the dark and germinated on custom-made ½ MS medium supplemented with 1 mM NH_4NO_3. Seedlings were grown under 80 µmol m^{-2} s^{-1} white light (L58W/830, Lumilux warm white, 5200 lm; Osram, Munich, Germany) at 22 °C in long-day conditions (16 h light/8 h dark) in MLR-351 growth chambers (Sanyo Denki, Eschborn, Germany). After 10 days, the primary root was excised below the first two emerging lateral roots, and plants were grown for an additional 3 days under the same conditions to generate split roots. Seedlings with two homogeneous main roots were then transferred to plates with two media compartments separated by a trench. Each side contained 18 mL of ½ MS medium supplemented with either 10 mM KNO_3 + 10 mM NH_4NO_3 (+N) or 10 mM KCl (mock). Treatments included: +N or mock on both sides (control), or +N on one side and mock on the other (split condition) to assess systemic nitrogen signaling. Plates were scanned 7 days after transfer at 600 dpi, and primary and lateral root lengths were quantified using Fiji (ImageJ). Total lateral root growth was calculated as the sum of all lateral root lengths divided by primary root length, as described previously (Ruffel et al., 2011). Statistical analyses and data visualization were performed in GraphPad Prism v10.

Confocal microscopy of the *Arabidopsis* shoot apical meristem. Confocal microscopy of *Arabidopsis* shoot apical meristems and image analysis were performed on plants at seven days after bolting. Meristems were excised from the inflorescence using Dumont No. 5 tweezers (Sigma, Taufkirchen, Germany) and placed on ½ MS medium 0.8% agarose for temporary storage before being dissected under a stereomicroscope (Olympus SZX16). SAMs were stained with 1.5 mM propidium iodide (PI) for 5 minutes after having been mounted on 2.5% agarose in 30 mm round plates, followed by washing with H_2O. Following a 15 min equilibration, z-stacks of SAMs were imaged with a step size of 0.7 µm in an Olympus FV1000 Laser Scanning Confocal Microscope using the Olympus 40x 0.80 NA LUMPlanFLN water immersion objective. Laser emission was set to 559 nm (PI). Confocal stacks were exported using

Fiji ImageJ, and SAM area was subsequently quantified using MorphoGraphX (Barbier de Reuille et al., 2015). Quantitative data were analyzed with GraphPadPrism (v10).

C/N quantification. For the determination of carbon and N content, in response to N treatments, *Arabidopsis* seeds were plated on 0.8% agarose N-free ½ MS supplemented with 1 mM NH_4NO_3 as the only N-source, stratified in the dark at 4 °C for 3 days, and grown for four days in a constant light regime at 80 µmol m^{-2} s^{-1} white light (L58W/830, Lumilux warm white, 5200 lm; Osram, Munich, Germany) at 22 °C. After four days seedlings were transferred to N-free ½ MS for two days as N-starvation conditions. Seedlings were subsequently transferred to 0.8% agarose N-free ½ MS supplemented with 10 mM NH_4NO_3 and 10 mM KNO_3 and grown for a further three days. Seedlings were collected in 2 mL Eppendorf tubes and dried at 65 °C for 2 days. 1 mg of dry weight per biological replicate was weighed and used for C/N quantification using a Carbon Nitrogen Elemental Analyzer. Quantitative data were analyzed with GraphPadPrism (v10).

Nitrate quantification. Nitrate quantification was performed on either entire *Arabidopsis* rosettes of four-week-old soil-grown plants or 11-day-old *Arabidopsis* seedlings using 30/40 mg of fresh weight plant material. Total nitrate content was quantified using the salicylic acid method as previously published (Cataldo et al., 1975). Quantitative data were analyzed with GraphPad Prism (v10).

Ammonium quantification. Ammonium quantification on 11-day-old *Arabidopsis* seedlings was performed using 30/40 mg of fresh weight plant material. Total ammonium content was quantified as previously described (Liu et al., 2021). Quantitative data were analyzed with GraphPad Prism (v10).

Nitrogen influx assays. For the determination of the N influx in *Physcomitrium*, colonies derived from a single gametophore inoculum were grown on normal BCDAT medium for four weeks at 22 °C with a 24-hour light period before being transferred to 4 mL of liquid N-free BCDAT medium (lacking KNO_3 and 5 mM $(NH_4)_2C_4H_4O_6$) in 12-well plates for 2 days. A total of three colonies were pooled together and considered as one biological replicate. After 2 days of N starvation, the plants' N response was induced by moving them to liquid BCDAT medium for 4 hours, rinsing with $CaSO_4$ for 1 minute, transferring to liquid medium containing 10 mM $^{15}NH_4{}^{15}NO_3$ (98% ^{15}N) for 12 minutes, and finally washing again with $CaSO_4$ for 1 minute before being collected and

frozen in liquid N. Prior to analysis, samples were dried at 70 °C for 4 days before the total ^{15}N and ^{14}N content was quantified from 0.7-1 mg of dried ground tissue using an elemental analyzer coupled with a mass spectroscope.

For the determination of the N influx in *Arabidopsis*, seeds were sterilized, plated on ½ MS containing 1 mM NH_4NO_3, and stratified for 2 days at 4 °C in the dark. Seedlings were grown for 11 days at 22 °C with a 24-hour light period before being transferred to 4 mL of liquid N-free ½ MS medium in 12-well plates. Around 12/15 seedlings were pooled together and considered as one biological replicate. After 2 days of N starvation, the plants' N response was induced by moving them to ½ MS containing 10 mM KNO_3 + 10 mM NH_4NO_3 medium for 4 hours, rinsing with $CaSO_4$ for 1 minute, transferring to liquid medium containing 10 mM $K^{15}NO_3$ (98% ^{15}N) + 10 mM $^{15}NH_4{}^{15}NO_3$ (98% ^{15}N) for 10 minutes, and finally washing again with $CaSO_4$ for 1 minute before being collected and frozen in liquid N. Prior to analysis, samples were dried at 70 °C for 4 days before the total ^{15}N and ^{14}N content was quantified from 0.7-1 mg of dried ground tissue using an elemental analyzer coupled with a mass spectroscope.

In both species, N influx was calculated by considering two fractions: N taken up before labelling ($f_{unlab-N}$) and N taken up during labelling (f_{lab-N}), with the relationship $f_{unlab-N} + f_{lab-N} = 1$. The f_{lab-N} was determined through isotopic mass balance as: $f_{lab-N} = \frac{atom\%^{15}N_{sample} - atom\%^{15}N_{unlab-N}}{atom\%^{15}N_{lab-N} - atom\%^{15}N_{unlab-N}}$; where: (I) $atom\%^{15}N_{sample}$ is the N isotopic composition of the labelled sample, (II) $atom\%^{15}N_{unlab-N}$ is the composition before labelling (from samples not exposed to the label), (III) $atom\%^{15}N_{lab-N}$ is the composition of the labelling N source (98% ^{15}N). The N influx (N_{influx}) during labelling, expressed as µmol g^{-1} DW h^{-1}, was then calculated as: $N_{influx} = f_{lab-N} * N_{total}$; where N_{total} is the total amount of N in the sample.

Sample preparation for RNA-seq. For RNA-seq analysis from *Physcomitrium*, freshly homogenized protonemata were grown at 22 °C for 4 days under continuous white light at 80 µmol m^{-2} s^{-1} (L58W/830, Lumilux warm white, 5200 lm; Osram, Munich, Germany). Plants were initially grown on cellophane discs positioned on 0.8% plant agar prepared with BCDAT medium containing 10 mM KNO_3 and 5 mM $(NH_4)_2C_4H_4O_6$ as N sources before transferring cellophane discs containing the protonemata for 2 days to N-free BCDAT medium, lacking KNO_3 and 5 mM $(NH_4)_2C_4H_4O_6$. For N-treatment and a corresponding N-free mock treatment,

cellophane discs containing the protonemata were transferred to 0.8% plant agar prepared with BCDAT medium containing 10 mM KNO_3 and 5 mM $(NH_4)_2C_4H_4O_6$ as N sources (+N, N-treatment) or BCDAT lacking KNO_3 and 5 mM $(NH_4)_2C_4H_4O_6$ but containing 10 mM KCl (-N, mock treatment). After 1 and 4 hours, protonemata were harvested and frozen in liquid N_2. RNA was subsequently extracted from 50-100 mg of plant material using the NucleoSpin RNA kit (Macherey-Nagel, Düren, Germany). Three biological replicate samples were prepared for each genotype.

For RNA-seq analysis of *Arabidopsis*, 50 mg of seeds per genotype were weighed and transferred to 2 mL Eppendorf tubes. Seeds were surface-sterilized with 25% commercial bleach (final concentration of 95 mM sodium hypochlorite) and 0.05% Triton X-100 for 7 min, then washed 4 times with sterile MilliQ water. Seeds were resuspended in 4 mL of sterile MilliQ, and 25 µL of seeds per well were plated in 12-well plates in 2 mL of customized N-free ½ MS (Biotrend Chemikalien, Cologne, Germany) liquid medium supplemented with 1 mM NH_4NO_3 as the only N source. Seeds were stratified in the dark at 4 °C for 3 days before being transferred to growth chambers in a constant light regime at 80 µmol m^{-2} s^{-1} white light (L58W/830, Lumilux warm white, 5200 lm; Osram, Munich, Germany) at 22 °C. Plants were cultivated in these conditions for 8 days, with a renewal of the growth medium after 4 days, before transferring the seedlings to 2 mL of N-free ½ MS for another two days for N starvation. After 2 days of N-starvation, the media were replaced by the N-free ½ MS supplemented with 10 mM NH_4NO_3 and 10 mM KNO_3 (+N, N-treatment) or N-free ½ MS supplemented with 10 mM KCl (-N, mock treatment). After 1 and 4 hours, plants were harvested and frozen in liquid N. RNA was subsequently extracted from 50-100 mg of plant material using the NucleoSpin RNA kit (Macherey-Nagel, Düren, Germany). Three biological replicate samples were prepared for each genotype.

RNA-seq. The quality and quantity of the extracted RNA were validated with a Bioanalyzer 2100 Microfluidics System (Agilent Technologies, Santa Cruz, CA). A total of 2 µg of RNA per sample was sent to the Cologne Center for Genomics (Cologne, Germany) for cDNA library synthesis and polyA+ mRNA sequencing on an Illumina NovaSeq® 6000 using paired-end sequencing (2 x 150 bp *Physcomitrium*; 2 x 100 bp) and 30M reads per sample.

Paired-end RNA-seq reads were trimmed with Cutadapt v2.8 with a PolyA trimming function of 12 nucleotides (-a "A{12}") (Martin). Read quality was assessed with

MultiQC v1.12 before and after trimming. The trimmed reads were aligned to either the *Physcomitrium patens* v6.1 genome (Phytozome genome ID: 870) (Bi et al., 2024) or the *Arabidopsis thaliana TAIR10* genome (Phytozome genome ID: 167) (Lamesch et al., 2012) using STAR v2.7.3a (Dobin et al., 2013). Alignment quality was checked again with MultiQC v1.12. Quantifications of transcripts were obtained using FeatureCounts v2.0.0 (Liao et al., 2014). Downstream data analysis was performed in R v4.3.2, and differential expression analysis was conducted using the DESeq2 package (v1.42.1). Lowly expressed genes were filtered out from the FeatureCounts output prior to differential expression analysis by retaining genes with a total read count across samples greater than 50 (rowSums > 50). Genes with an adjusted p-value < 0.05 were considered significantly differentially expressed. The PCA was performed on the variance-stabilized transformed counts using the plotPCA function from the DESeq2 (v1.42.1) package. Volcano plots were generated using the EnhancedVolcano package (v 1.20.0) and heatmaps using the pheatmap package (v 1.0.12). Raw data of both experiments were deposited to the repository of NFDI4plants under Madland ARC Schwechheimer (http://git.nfdi4plants.org). Further information will be made available upon acceptance of the article.

Gene Ontology (GO) enrichment analysis. For GO enrichment analysis, *Physcomitrium* gene IDs were first converted to genome version v3.3 nomenclature using the conversion table (N° 20230906) available on PEATmoss (https://peatmoss.plantcode.cup.uni-freiburg.de/ppatens_db/downloads.php). GO enrichment analysis for *Physcomitrium* was performed using g:Profiler (Kolberg et al., 2023). GO enrichment for *Arabidopsis* was conducted in R using the clusterProfiler package (Wu et al., 2021).

SUPPLEMENTAL FIGURES

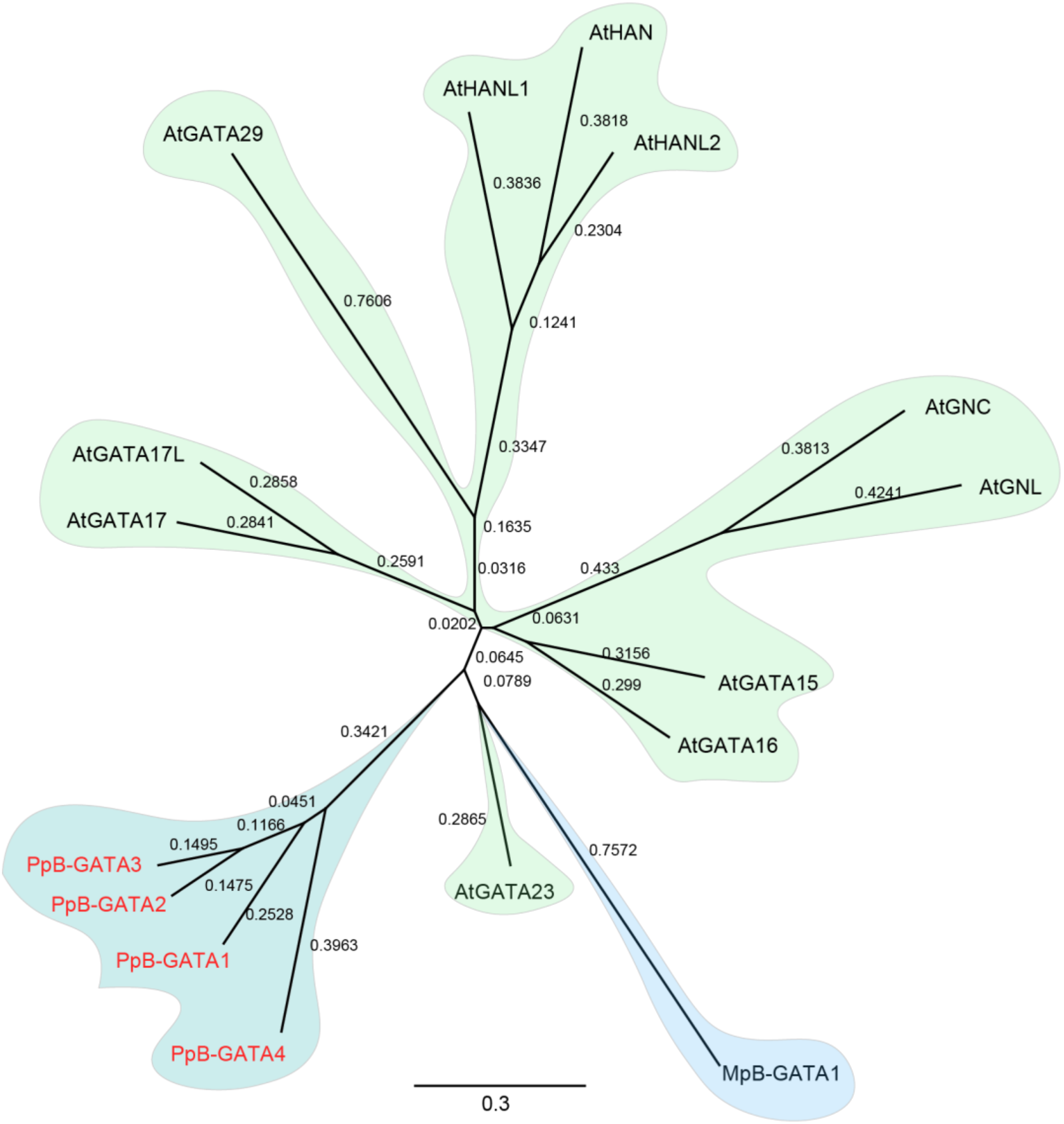

Supplemental Figure S1. Phylogenetic tree of B-GATA proteins from *Physcomitrium patens* (Pp), *Marchantia polymorpha* (Mp), and *Arabidopsis thaliana* (At). Phylogenetic tree of the B-GATA proteins from *Physcomitrium* (Pp), *Marchantia polymorpha* (Mp) and *Arabidopsis thaliana* (At) obtained by aligning the zinc finger domains using MUSCLE and tree construction using the Jukes-Cantor genetic distance model and the Neighbor-Joining tree build method. Numbers and the scale bar represent the evolutionary distance in terms of amino acid substitutions per site.

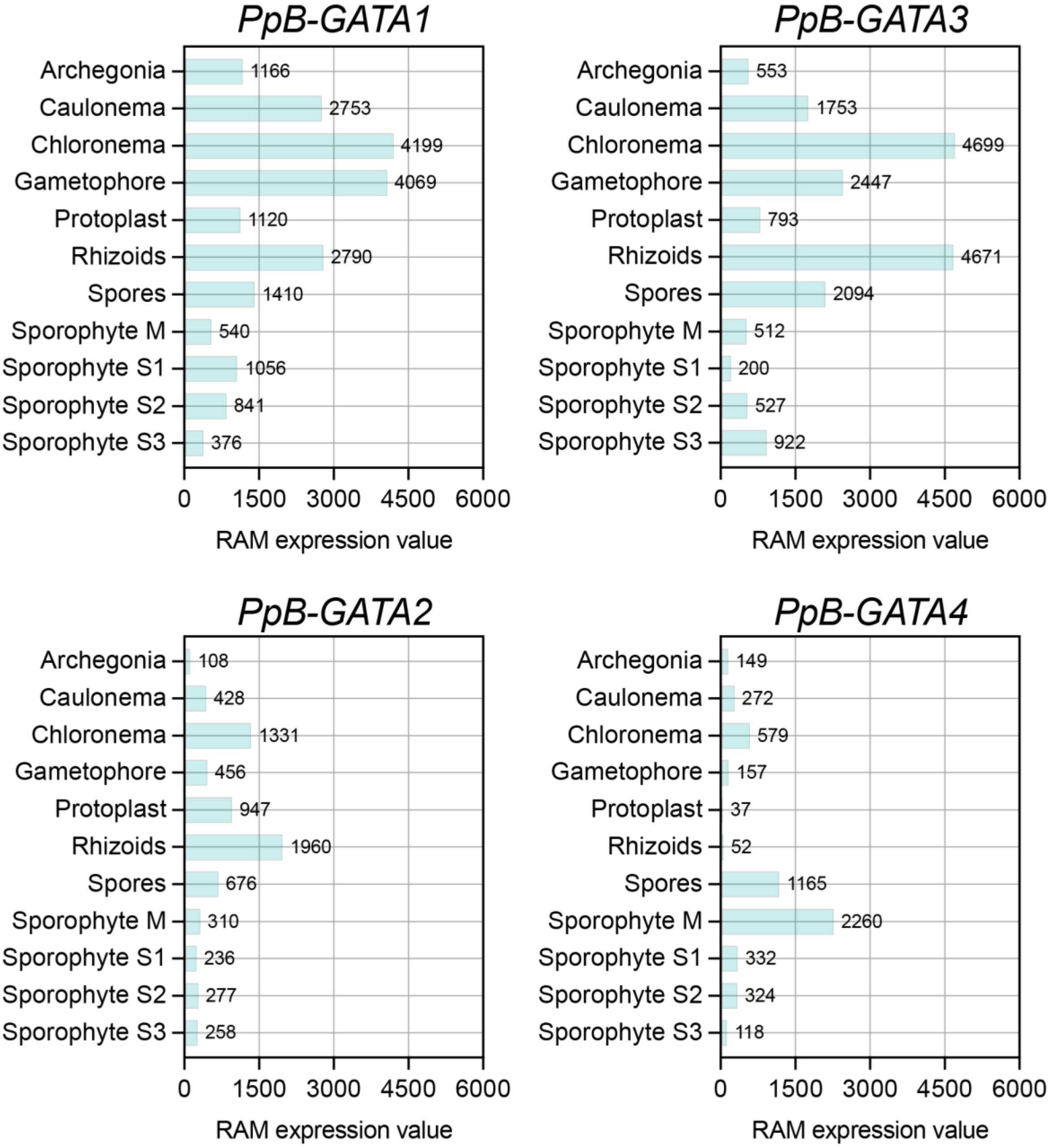

Supplemental Figure S2. *PpB-GATA* gene expression levels across different tissues in *Physcomitrium patens*. Graphs showing the relative absolute expression (RAM) values of the four Physcomitrium *B-GATAs* across different tissues, as specified and extracted from the eFP *Physcomitrium* browser (Winter et al., 2007; Ortiz-Ramirez et al., 2016).

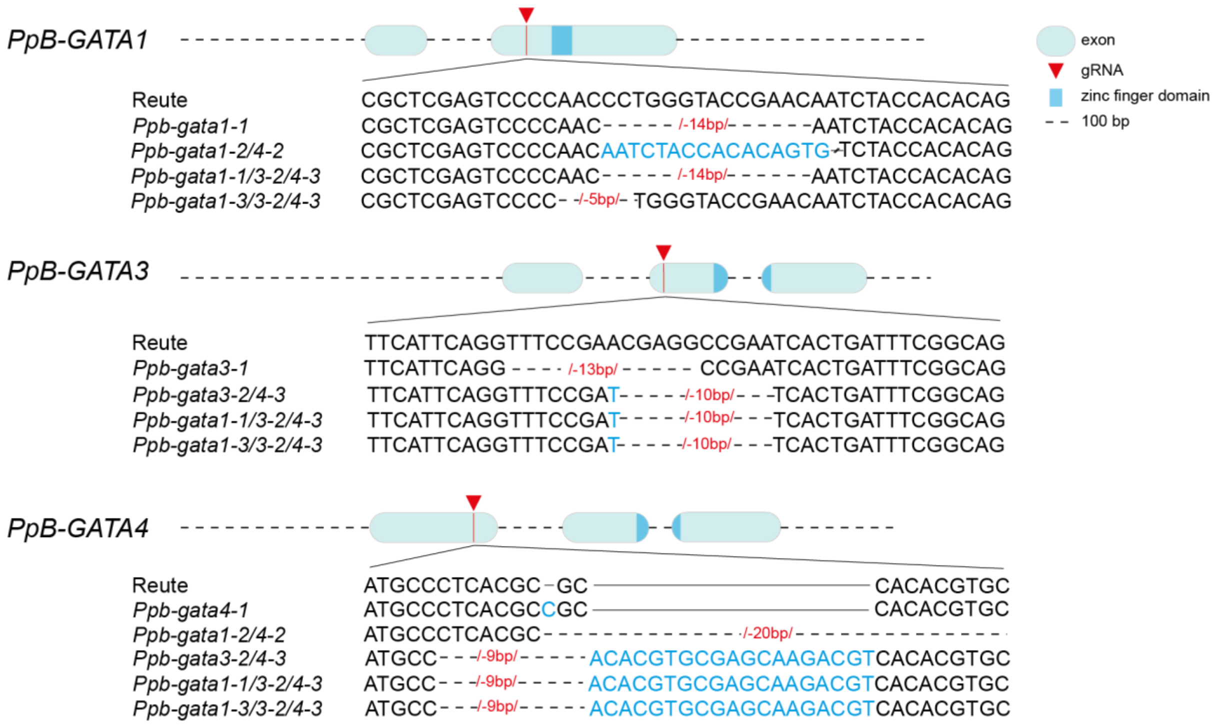

Supplemental Figure S3. Genotypes of *Physcomitrium patens Ppb-gata* mutant alleles obtained by CRISPR/Cas9-based mutagenesis. Graphical representation of the *PpB-GATA* genes *PpB-GATA1, PpB-GATA3, and PpB-GATA4* (dotted lines, introns; rounded rectangles, exons; turquoise elements, zinc finger). Positions of gRNAs used for mutagenesis are shown by the red arrowheads. Sequences of the Reute wild type and the respective mutant alleles in single, double, or triple mutants are displayed, with mutations being highlighted in red (deletions) or blue (insertions).

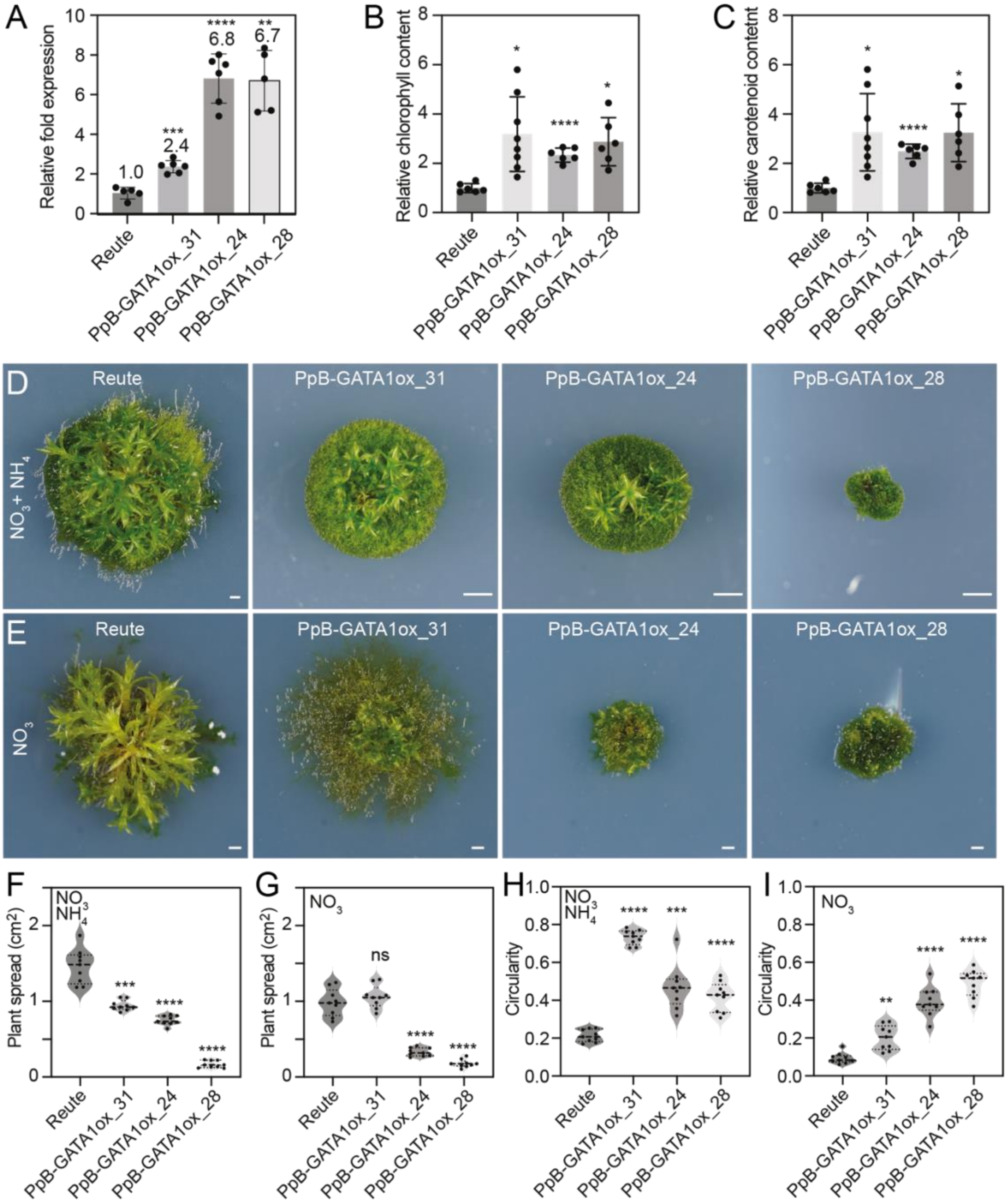

Supplemental Figure S4. *PpB-GATA1* overexpression suppresses growth and enhances chlorophyll and carotenoid accumulation in *Physcomitrium patens*. (A) Quantification of *PpB-GATA1* transcript levels in three independent overexpression lines relative to the Reute wild type. **(B, C)** Chlorophyll (chlorophyll a + b; **B**) and carotenoid contents **(C)** in the same lines shown, relative to the Reute wild type. **(D, E)** Representative images of 4-week-old plants of the indicated genotypes grown on medium containing either NO_3^- + NH_4^+ (D) or NO_3^- (E) as N sources. Scale bars = 1 mm. **(G)** Representative confocal images of calcofluor white-stained protonemal filaments. Arrows mark the division planes used for angle measurements in (F). Scale bar = 50 µm. **(F–I)** Quantification of plant spread area **(F, G)** and circularity **(H, I)** from plants shown in (D) and (E), respectively. Each data point

in (F-I) represents an independent biological replicate; horizontal black lines indicate the median, and dotted lines mark the first (Q1) and third (Q3) quartiles. Brown-Forsythe and Welch ANOVA followed by Dunnett's T3 multiple comparisons test, with the Reute wild type as a control. *, $p < 0.05$; **, $p < 0.01$; ***, $p < 0.001$; ****, $p < 0.0001$; ns, not significant.

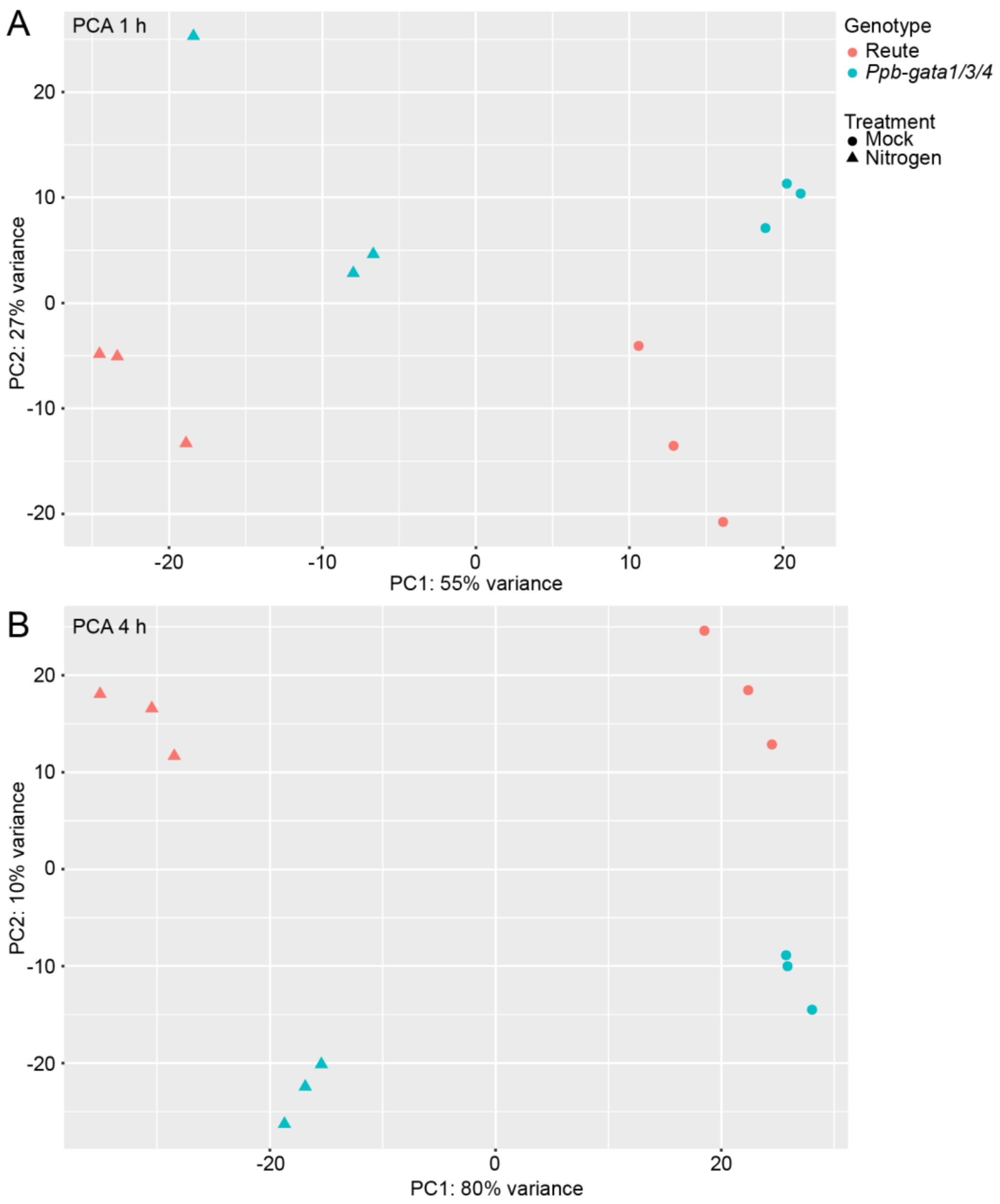

Supplemental Figure S5. Principal component analysis (PCA) of transcriptomic profiles from *Physcomitrium patens* Reute and the *Ppb-gata1/3/4* triple mutant. (A, B) PCA scatter plots showing the variance in gene expression among three biological replicates of Reute (wild type) and the *Ppb-gata1/3/4* triple mutant at 1 hour (A) and 4 hours (B) after N or a corresponding mock treatment. Each data point represents one replicate.

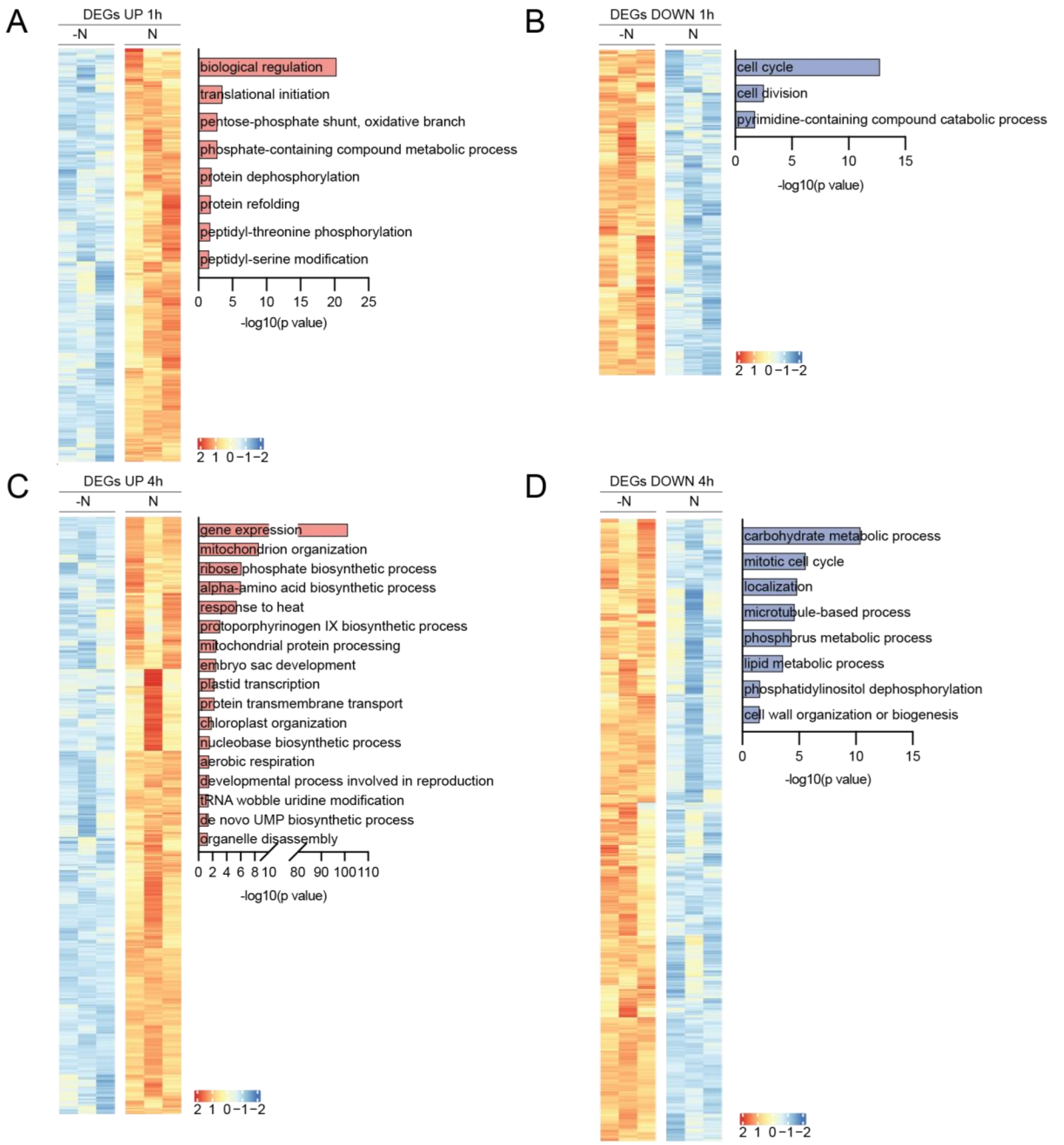

Supplementary Figure S6. Gene ontology (GO) enrichment analysis of differentially expressed genes (DEGs) in response to nitrogen in *Physcomitrium patens* Reute. (A–D) Heatmaps showing clustered expression profiles of DEGs from N-treated (N) and mock-treated (–N) plants at the 1-hour (A, B) and 4-hour (C, D) time points. DEGs are grouped into upregulated (A, C) and downregulated (B, D) gene sets. Color intensity represents relative gene expression levels normalized by centering each row to its mean expression across all samples. Significantly enriched GO categories ($p < 0.05$, determined using g:Profiler) are shown to the right of each cluster.

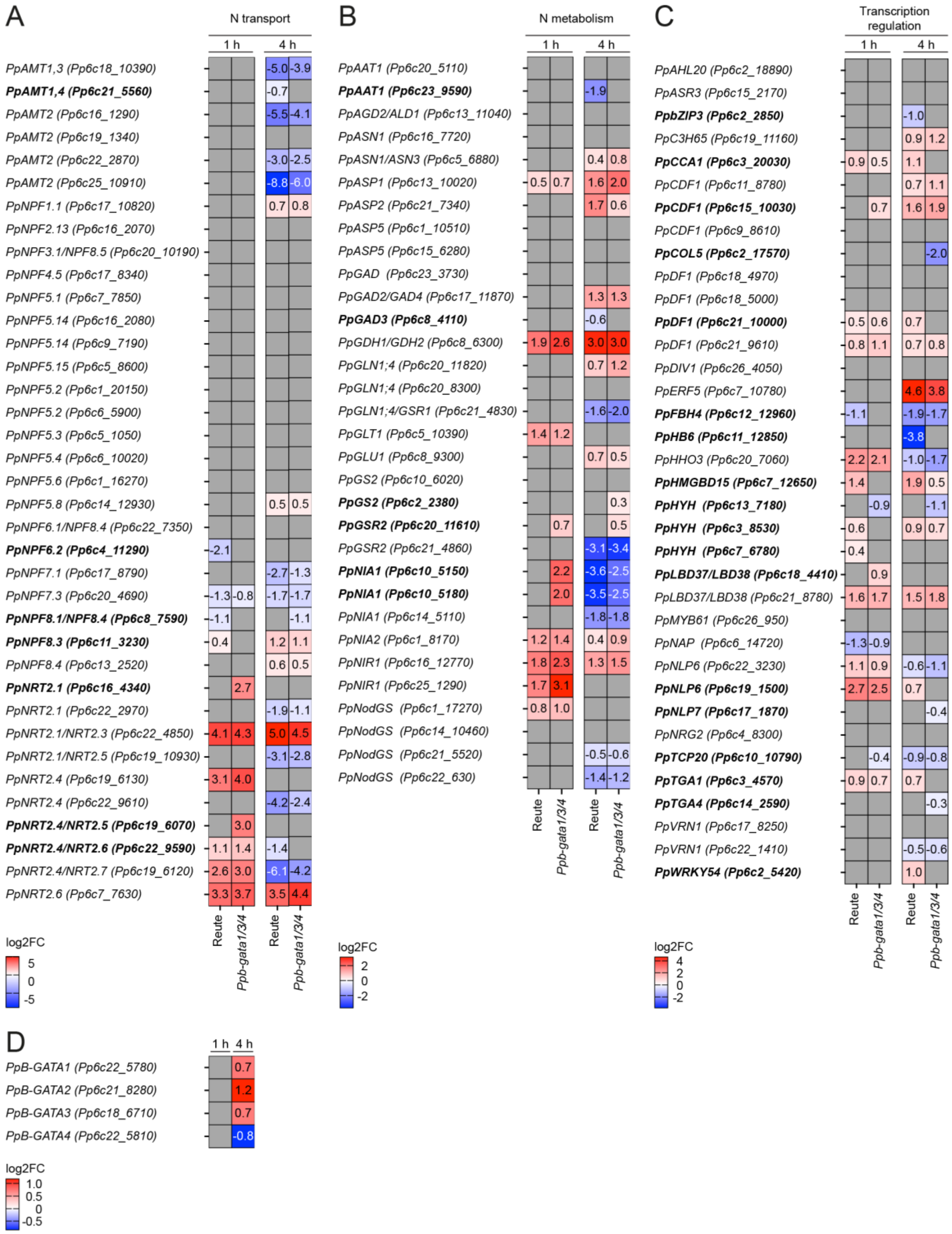

Supplemental Figure S7. Differential regulation of nitrogen transport, metabolism, and transcription regulation genes may underlie the reduced N response in *Physcomitrium patens Ppb-gata1/3/4* mutants. (A–C) Heat maps showing $\log_2$ fold change ($\log_2$FC) in gene expression for genes involved in N transport (A), N metabolism (B), and N-responsive transcriptional regulation (C) in Reute wild type and Ppb-gata1/3/4 mutants at 1-hour and 4-hour time points following N treatment or

the corresponding mock condition. The list of *Physcomitrium* orthologous genes is provided in Supplemental Table S7. **(D)** Heat map showing log_2FC expression changes of the four *PpB-GATA* genes under the same conditions. Log_2FC values are derived from DESeq2 analysis ($p < 0.05$) and color-coded as indicated in the legend. Grey boxes represent genes with non-significant changes (adjusted p-value > 0.05). Genes showing a differential response in the *Ppb-gata1/3/4* mutant in comparison to the wild type are highlighted in bold.

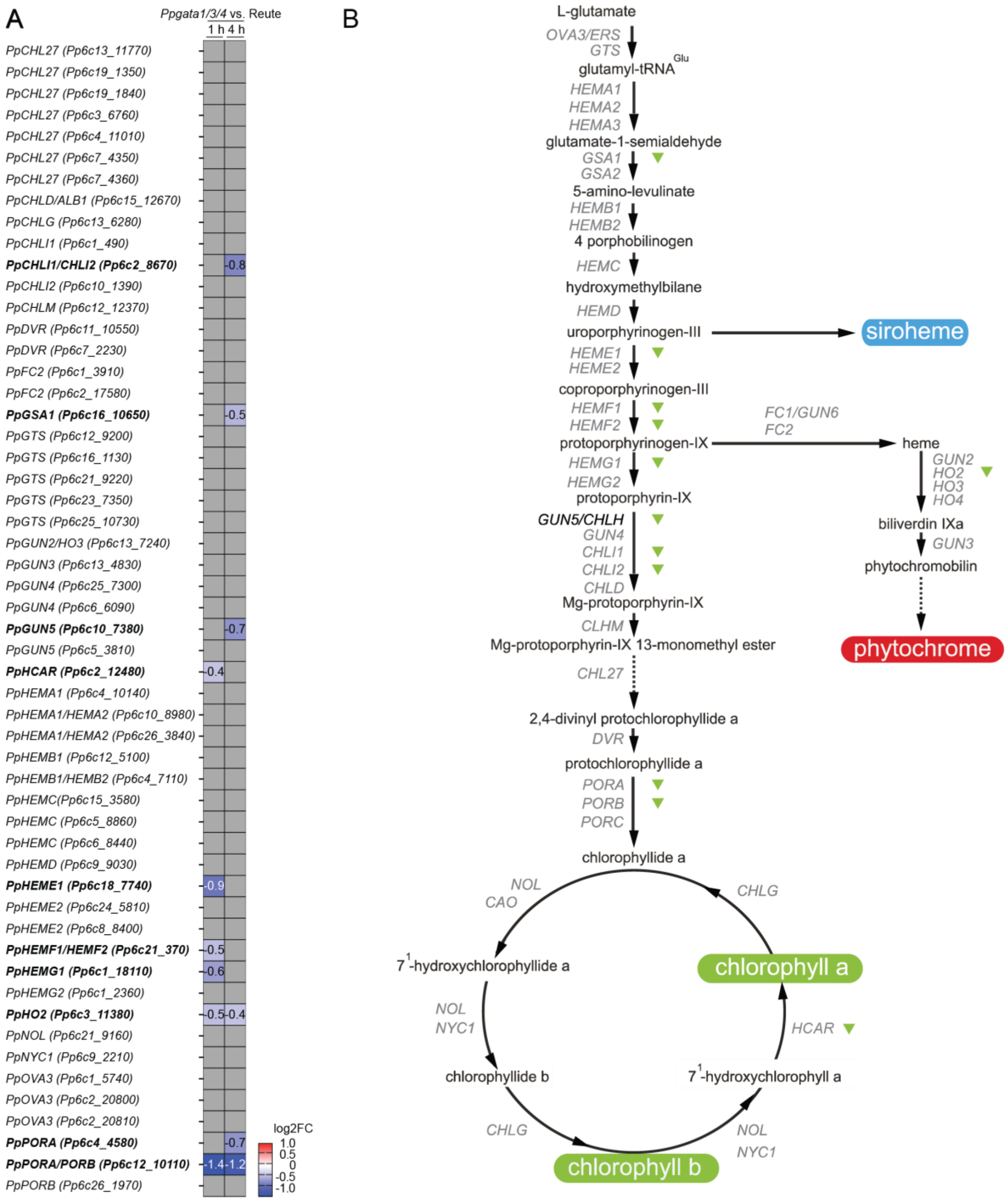

Supplemental Figure S8. Altered expression of chlorophyll biosynthesis genes may contribute to reduced chlorophyll accumulation in *Physcomitrium patens Ppb-gata1/3/4* mutants. (A) Heat map showing $\log_2$ fold change ($\log_2$FC) in the expression of chlorophyll biosynthesis genes in *Ppb-gata1/3/4* compared to the Reute wild type under mock conditions at the 1-hour and 4-hour time points. Log_2FC values are based on DESeq2 analysis ($p < 0.05$) and are color-coded according to the legend. Grey boxes indicate genes with non-significant expression changes (adjusted p-value > 0.05). The list of *Physcomitrium* orthologous genes is provided in Supplemental Table S7. **(B)** Schematic

representation of the *Arabidopsis* chlorophyll biosynthesis pathway, adapted from (Bastakis et al., 2018). Green arrowheads indicate genes whose *Physcomitrium* orthologs are downregulated in *Ppb-gata1/3/4* as shown in (A). Genes showing a differential response in the *Ppb-gata1/3/4* mutant in comparison to the wild type are highlighted in bold.

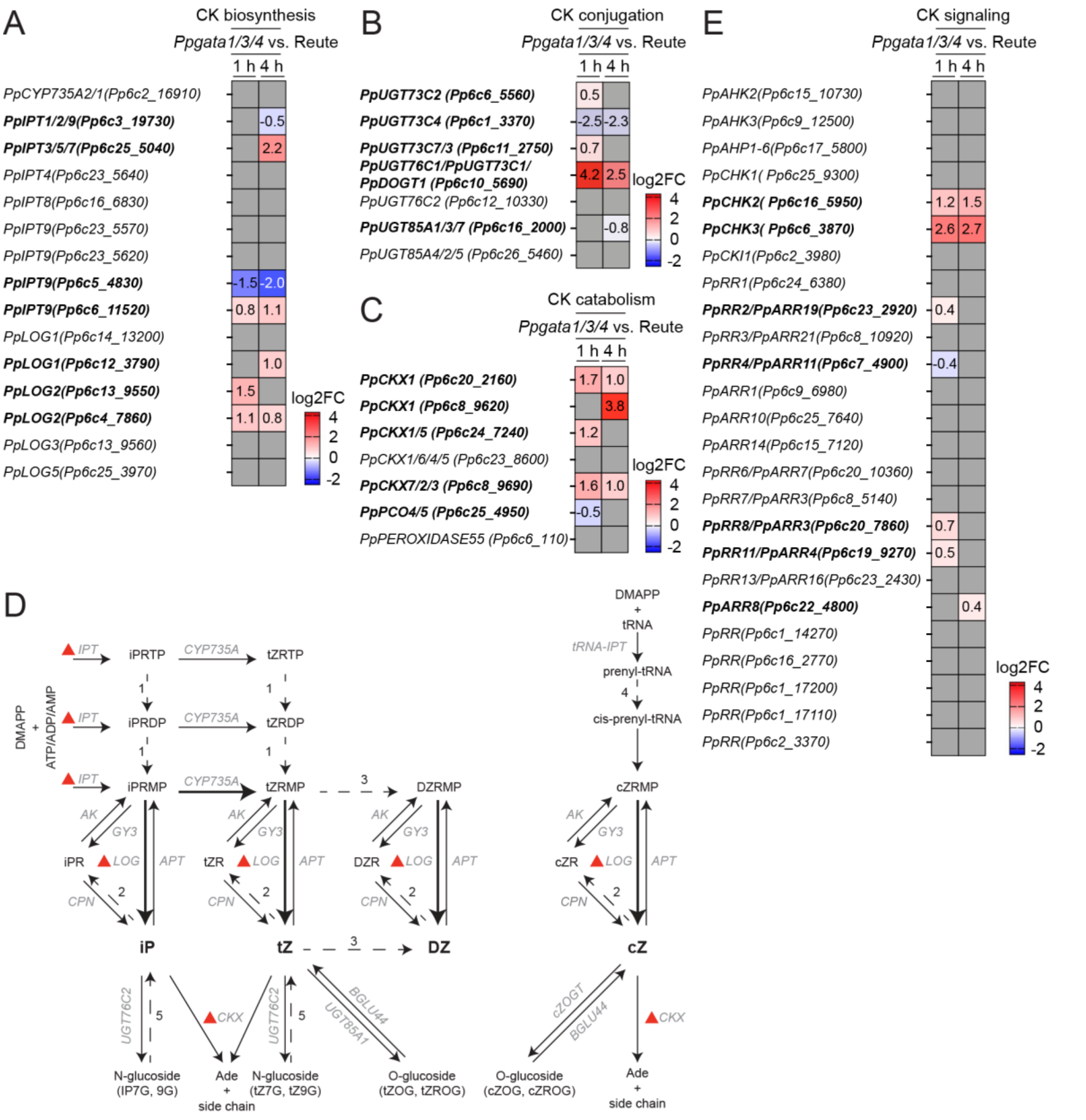

Supplemental Figure S9. Expression profiles of cytokinin biosynthesis genes in *Physcomitrium patens* Ppb-gata1/3/4 mutants. (A-C, E) Heat maps showing $\log_2$ fold change ($\log_2$FC) in the expression of cytokinin biosynthesis genes in the categories cytokinin biosynthesis (A), cytokinin conjugation (B), cytokinin catabolism (C) and cytokinin signaling (E) in *Ppb-gata1/3/4* compared to the Reute wild type under mock conditions at the 1-hour and 4-hour time points. Log_2FC values are based on DESeq2 analysis ($p < 0.05$) and are color-coded according to the legend. Grey boxes indicate genes with non-significant expression changes (adjusted p-value > 0.05). The list of *Physcomitrium* orthologous genes is provided in Supplemental Table S7. Genes showing a differential response in the *Ppb-gata1/3/4* mutant in comparison to the wild type are highlighted in bold. **(D)** Schematic model of the cytokinin biosynthesis pathway in plants, adapted from (Sakakibara, 2025). Red arrowheads mark genes that are upregulated in the RNA-seq data set that may explain the cytokinin accumulation

observed in the *Ppb-gata1/3/4* mutant as shown in (A) – (C). Abbreviations: IPT (adenosine phosphate-isopentenyltransferase), DMAPP (dimethylallyl diphosphate), AK (adenosine kinase), APT (adenine phosphoribosyltransferase), CYP735A (cytochrome P450 monooxygenase 735A), LOG (LONELY GUY), GY3 (5'-ribonucleotide phosphohydrolase GRAIN YIELD3), CPN (cytokinin/purine riboside nucleosidase), CKX (cytokinin oxidase), UGT85A1 (O-glucosyltransferase UGT85A1), BGLU44 (β-glucosidase BGLU44), and UGT76C2 (N-glucosyltransferase UGT76C2), 1. phosphatase, 2, purine nucleoside phosphorylase; 3, zeatin reductase; 4, cis-hydroxylase; 5, tentatively a β-glucosidase.

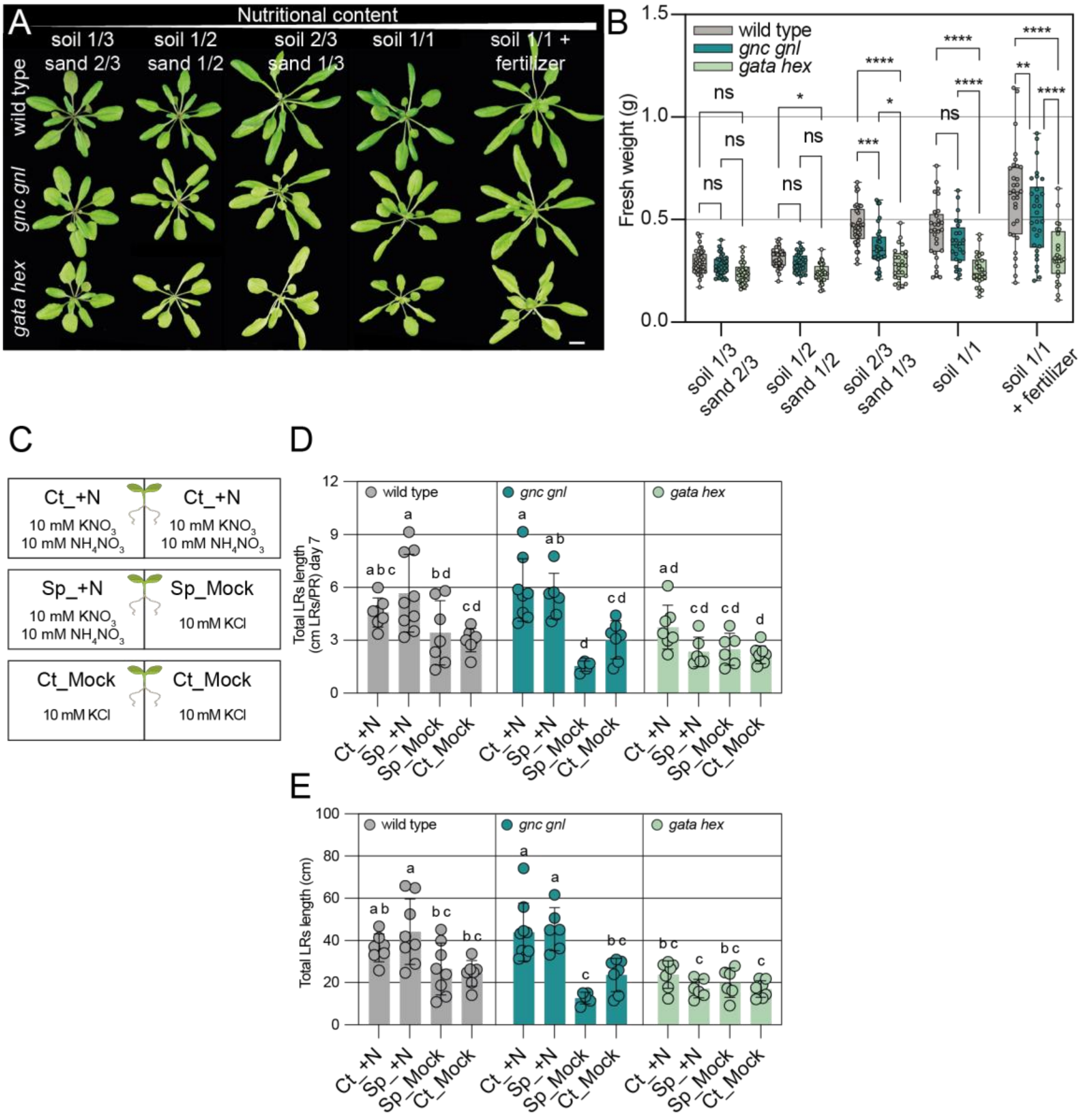

Supplemental Figure S10. Loss of *GNC* and *GNL* leads to a partial suppression of N-dependent growth responses in *Arabidopsis thaliana*. (A) Representative images of four-week-old *Arabidopsis* wild type (Col-0), *gnc gnl*, and *gata hexuple* (*gata hex*) mutant plants, grown under varying soil/sand mixtures or soil supplemented with fertilizer. Scale bar = 1 cm. **(B)** Quantification of fresh weight in *Arabidopsis* plants as shown in (A). Each data point represents an independent biological replicate (one entire rosette); the whiskers indicate the full data range (minimum to maximum), boxes show the interquartile range (IQR; spanning the 25th to 75th percentiles), and the central line indicates the median value of the data distribution. Two-way ANOVA comparisons test, followed by Tukey's multiple comparisons performed for each soil and sand combination: * $p < 0.05$, ** $p < 0.01$, *** $p < 0.001$, **** $p < 0.0001$, ns: not significant. **(C)** Schematic representation of the split-root setup used for the analysis shown in (D, E). Roots were subjected to three conditions: Ct_+N (control + nitrogen) and Ct_Mock (control mock), where both sides of the root system were exposed to either +N or mock treatment, respectively; and Split_+N/Sp_Mock, where one side was supplied with N and the other with mock (KCl). **(D, E)** Total lateral root (LR) growth in wild-type Col-0, *gnc gnl*, and *gata hex* under the conditions

described in (C). In (D), total LR length is normalized to primary root (PR) length; in (E), absolute total LR length is shown. Each point represents an independent biological replicate; bars indicate mean ± SD. Different letters denote statistically significant differences (two-way ANOVA followed by Tukey's multiple comparisons test).

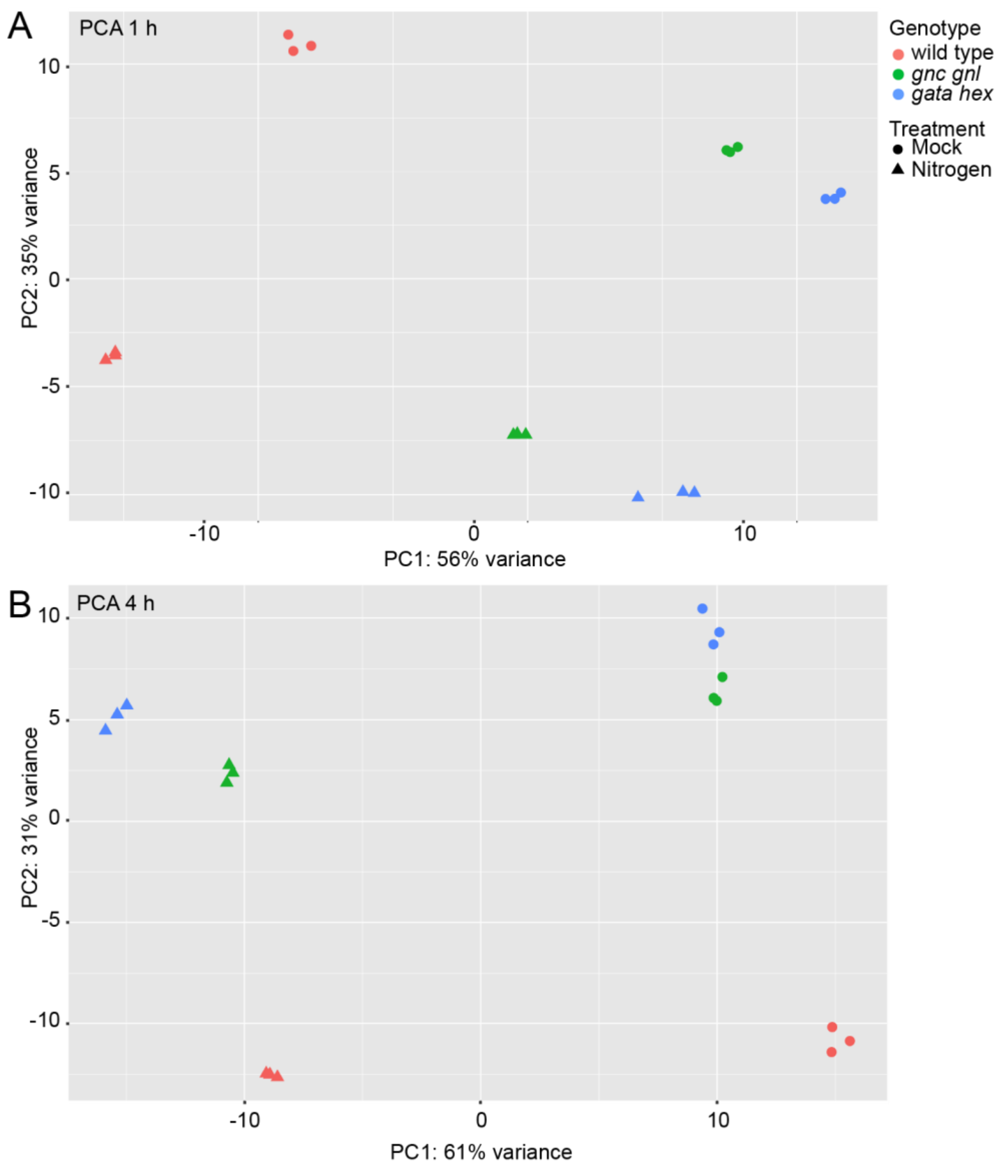

Supplemental Figure S11. Principal component analysis (PCA) of transcriptomic profiles from *Arabidopsis thaliana* and *b-gata* mutants. (A, B) PCA scatter plots showing the variance in gene expression among three biological replicates of *Arabidopsis* wild type, *gnc gnl* and the *gata hexuple* (*gata hex*) at 1 hour (A) and 4 hours (B) after N or a corresponding mock treatment. Each data point represents one replicate.

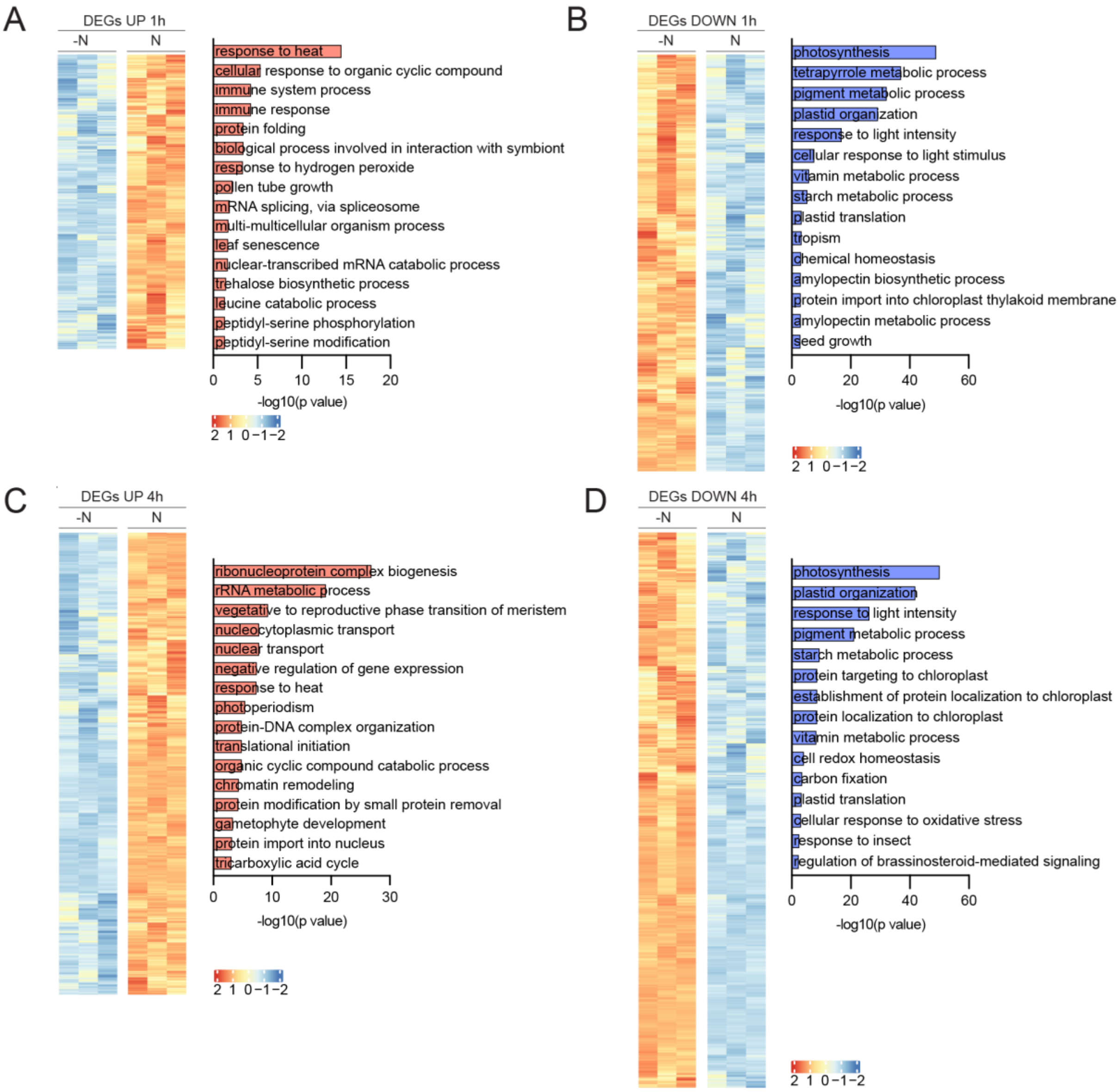

Supplemental Figure S12. Gene ontology (GO) enrichment analysis of differentially expressed genes (DEGs) in response to N in *Arabidopsis thaliana*. (A–D) Heatmaps showing clustered expression profiles of DEGs from N-treated (N) and mock-treated (–N) plants at the 1-hour (A, B) and 4-hour (C, D) time points. DEGs are grouped into upregulated (A, C) and downregulated (B, D) gene sets. Color intensity represents relative gene expression levels normalized by centering each row to its mean expression across all samples. Significantly enriched GO categories ($p < 0.05$, determined using ClusterProfiler) are shown to the right of each cluster.

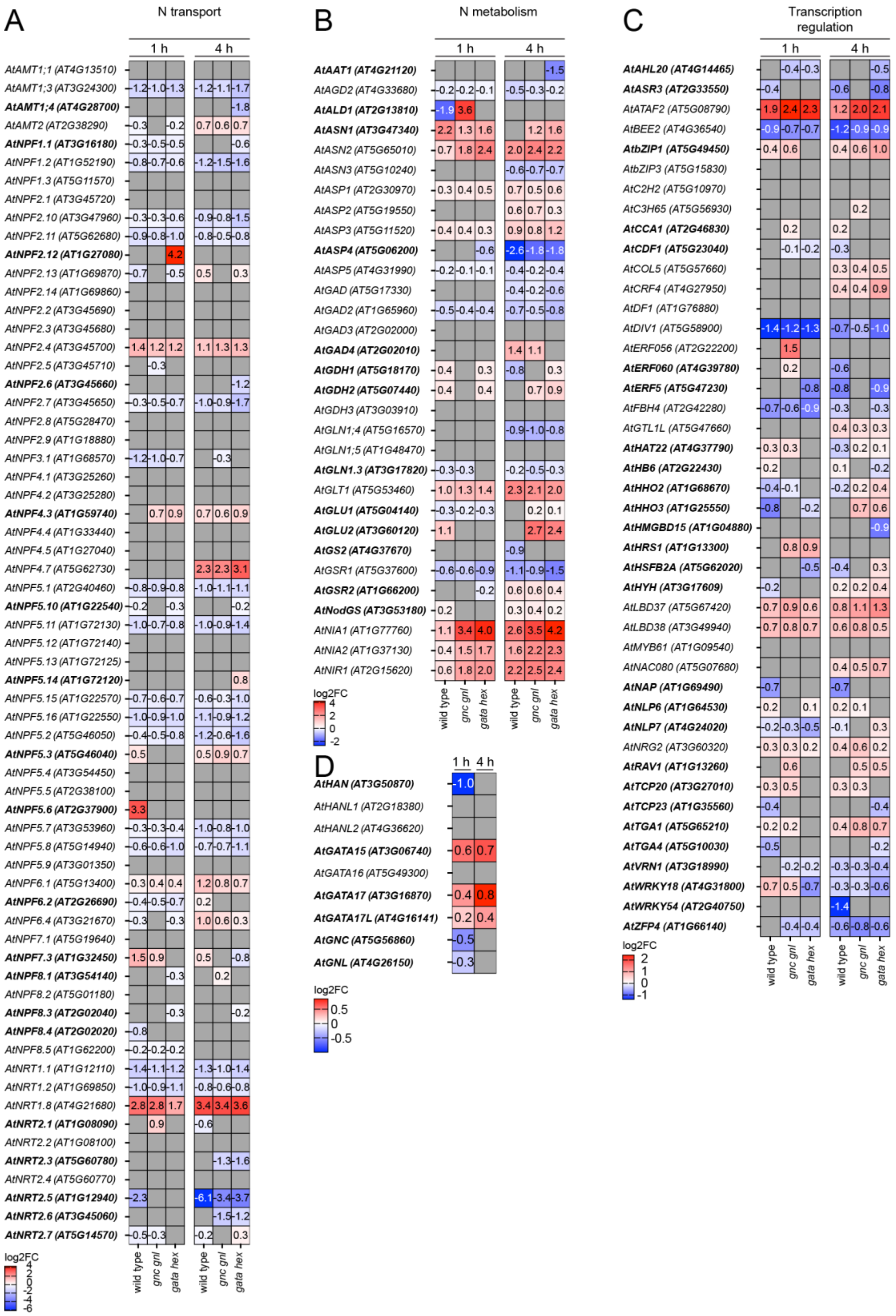

Supplemental Figure S13. Differential regulation of nitrogen transport, metabolism, and transcription regulation genes may underlie the reduced N response in *gnc gnl* and *gata hex* mutants from *Arabidopsis thaliana*. (A–C) Heat maps showing $\log_2$ fold change ($\log_2$FC) in gene

expression for genes involved in N transport (A), N metabolism (B), and N-responsive transcription regulation (C) in the wild type and *gnc* gnl or *gata* hex mutants at the 1-hour and 4-hour time points following N treatment or the corresponding mock condition. **(D)** Heat map showing log_2FC expression changes of the three HAN-domain and the six *bona fide* LLM-domain *B-GATA* genes at the 1-hour and 4-hour time points following N treatment. Log_2FC values are derived from DESeq2 analysis ($p < 0.05$) and color-coded as indicated in the legend. Grey boxes represent genes with non-significant changes (adjusted p-value > 0.05). Genes showing a differential N-response in the *gata hex* mutant compared to the wild type are highlighted in bold.

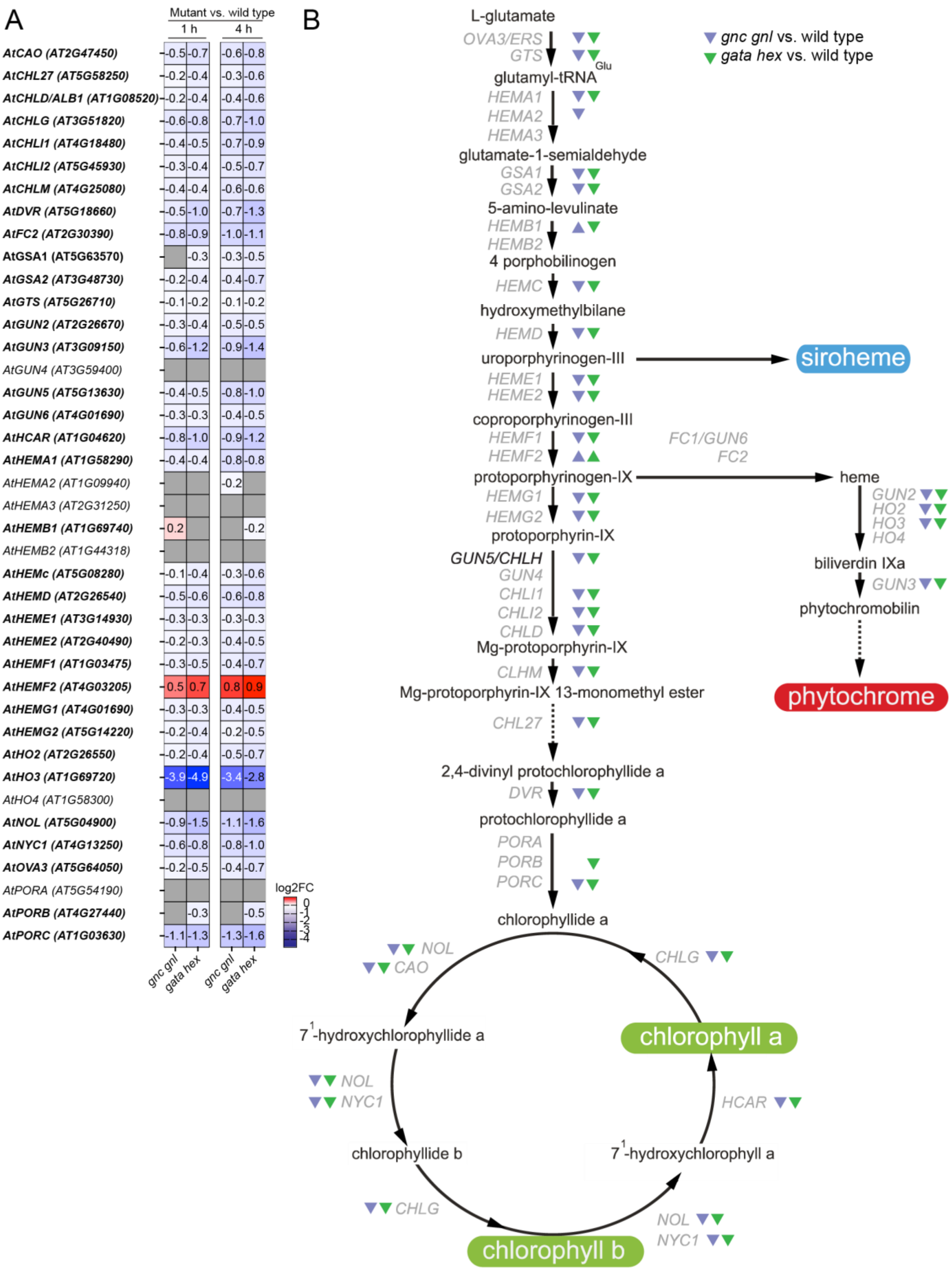

Supplemental Figure S14. Altered expression of chlorophyll biosynthesis genes in *Arabidopsis thaliana gnc gnl* and *gata hex* mutants. (A) Heat map showing log_2 fold change (log_2FC) in the expression of chlorophyll biosynthesis genes in *gnc gnl* and *gata hex* compared to the *Arabidopsis* wild type under mock conditions at the 1-hour and 4-hour time points. Log_2FC values are based on DESeq2

analysis ($p < 0.05$) and are color-coded according to the legend. Grey boxes indicate genes with non-significant expression changes (adjusted p-value > 0.05). The list of *Arabidopsis* chlorophyll biosynthesis genes is provided in Supplemental Table S7. Genes showing a differential N-response in the *gata hex* mutant compared to the wild type are highlighted in bold. **(B)** Schematic representation of the *Arabidopsis* chlorophyll biosynthesis pathway, adapted from (Bastakis et al., 2018). Light blue and green arrowheads indicate genes downregulated in *gnc gnl* and, respectively, *gata hex* as shown in (A). Genes showing a differential response in the *gata hex* mutant in comparison to the wild type are highlighted in bold.

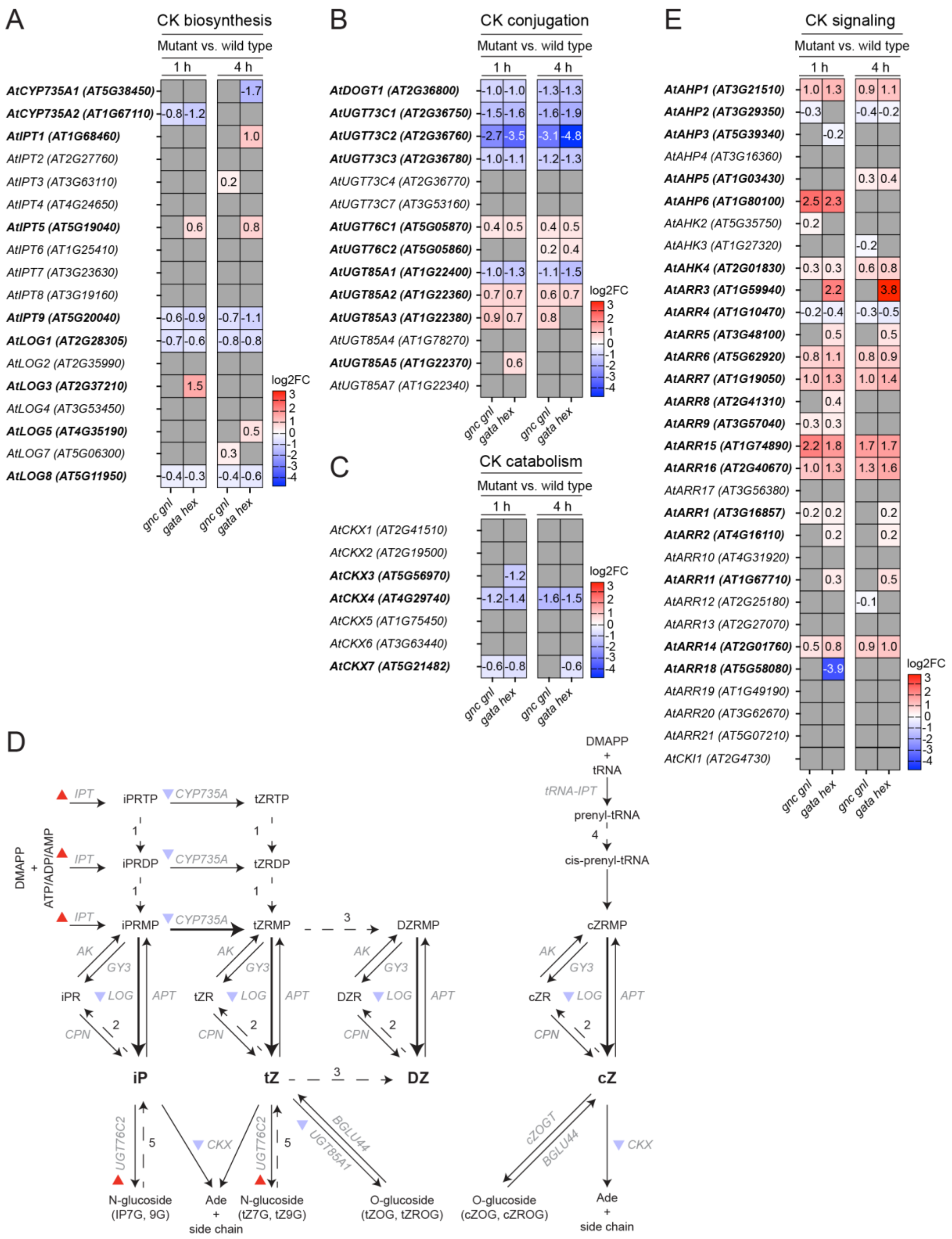

Supplemental Figure S15. Expression profiles of cytokinin biosynthesis genes in *Arabidopsis thaliana gnc gnl* and *gata hex* mutants. (A-C, E) Heat maps showing $\log_2$ fold change ($\log_2$FC) in the expression of cytokinin biosynthesis genes in the categories cytokinin biosynthesis (A), cytokinin conjugation (B), cytokinin catabolism (C), and cytokinin signaling (E) in *gnc* gnl and *gata* hex compared to the *Arabidopsis* wild type under mock conditions at the 1-hour and 4-hour time points. Log_2FC values

are based on DESeq2 analysis ($p < 0.05$) and are color-coded according to the legend. Grey boxes indicate genes with non-significant expression changes (adjusted p-value > 0.05). The list of *Arabidopsis* genes is provided in Supplemental Table S7. Genes showing a differential response in the *gata hex* mutant in comparison to the wild type are highlighted in bold. **(D)** Schematic model of isoprenoid cytokinin biosynthesis pathway in plants, adapted from (Sakakibara, 2025). Red and blue arrowheads mark genes that are differentially regulated in the RNA-seq data set and may explain cytokinin accumulation observed in *gata hex*. Abbreviations: IPT (adenosine phosphate-isopentenyltransferase), DMAPP (dimethylallyl diphosphate), AK (adenosine kinase), APT (adenine phosphoribosyltransferase), CYP735A (cytochrome P450 monooxygenase 735A), LOG (LONELY GUY), GY3 (5'-ribonucleotide phosphohydrolase GRAIN YIELD3), CPN (cytokinin/purine riboside nucleosidase), CKX (cytokinin oxidase), UGT85A1 (O-glucosyltransferase UGT85A1), BGLU44 (β-glucosidase BGLU44), and UGT76C2 (N-glucosyltransferase UGT76C2), 1. phosphatase, 2, purine nucleoside phosphorylase; 3, zeatin reductase; 4, cis-hydroxylase, 5, tentatively a β-glucosidase.

SUPPLEMENTAL REFERENCES

Ashton NW, Grimsley NH, Cove DJ (1979) Analysis of gametophytic development in the moss, Physcomitrella patens, using auxin and cytokinin resistant mutants. Planta **144:** 427–435

Barbier de Reuille P, Routier-Kierzkowska AL, Kierzkowski D, Bassel GW, Schupbach T, Tauriello G, Bajpai N, Strauss S, Weber A, Kiss A, Burian A, Hofhuis H, Sapala A, Lipowczan M, Heimlicher MB, Robinson S, Bayer EM, Basler K, Koumoutsakos P, Roeder AH, Aegerter-Wilmsen T, Nakayama N, Tsiantis M, Hay A, Kwiatkowska D, Xenarios I, Kuhlemeier C, Smith RS (2015) MorphoGraphX: A platform for quantifying morphogenesis in 4D. Elife **4:** 05864

Bastakis E, Hedtke B, Klermund C, Grimm B, Schwechheimer C (2018) LLM-Domain B-GATA Transcription Factors Play Multifaceted Roles in Controlling Greening in Arabidopsis. Plant Cell **30:** 582–599

Bi G, Zhao S, Yao J, Wang H, Zhao M, Sun Y, Hou X, Haas FB, Varshney D, Prigge M, Rensing SA, Jiao Y, Ma Y, Yan J, Dai J (2024) Near telomere-to-telomere genome of the model plant Physcomitrium patens. Nat Plants **10:** 327–343

Cataldo D, Maroon M, Schrader L, Youngs V (1975) Rapid colorimetric determination of nitrate in plant tissue by nitration of salicylic acid. *In* Communications in soil science and plant analysis,

Cingolani P, Platts A, Wang le L, Coon M, Nguyen T, Wang L, Land SJ, Lu X, Ruden DM (2012) A program for annotating and predicting the effects of single nucleotide polymorphisms, SnpEff: SNPs in the genome of Drosophila melanogaster strain w1118; iso-2; iso-3. Fly (Austin) **6:** 80–92

Collonnier C, Epert A, Mara K, Maclot F, Guyon-Debast A, Charlot F, White C, Schaefer DG, Nogue F (2017) CRISPR-Cas9-mediated efficient directed mutagenesis and RAD51-dependent and RAD51-independent gene targeting in the moss Physcomitrella patens. Plant Biotechnol J **15:** 122–131

Coudert Y, Novak O, Harrison CJ (2019) A KNOX-Cytokinin Regulatory Module Predates the Origin of Indeterminate Vascular Plants. Curr Biol **29:** 2743–2750 e2745

Danecek P, Bonfield JK, Liddle J, Marshall J, Ohan V, Pollard MO, Whitwham A, Keane T, McCarthy SA, Davies RM, Li H (2021) Twelve years of SAMtools and BCFtools. Gigascience **10**

Dobin A, Davis CA, Schlesinger F, Drenkow J, Zaleski C, Jha S, Batut P, Chaisson M, Gingeras TR (2013) STAR: ultrafast universal RNA-seq aligner. Bioinformatics **29:** 15–21

Jiang H, Lei R, Ding SW, Zhu S (2014) Skewer: a fast and accurate adapter trimmer for next-generation sequencing paired-end reads. BMC Bioinformatics **15:** 182

Kolberg L, Raudvere U, Kuzmin I, Adler P, Vilo J, Peterson H (2023) g:Profiler-interoperable web service for functional enrichment analysis and gene identifier mapping (2023 update). Nucleic Acids Res **51:** W207–W212

Lamesch P, Berardini TZ, Li D, Swarbreck D, Wilks C, Sasidharan R, Muller R, Dreher K, Alexander DL, Garcia-Hernandez M, Karthikeyan AS, Lee CH, Nelson WD, Ploetz L, Singh S, Wensel A, Huala E (2012) The Arabidopsis Information Resource (TAIR): improved gene annotation and new tools. Nucleic Acids Res **40:** D1202–1210

Lampropoulos A, Sutikovic Z, Wenzl C, Maegele I, Lohmann JU, Forner J (2013) GreenGate---a novel, versatile, and efficient cloning system for plant transgenesis. PLoS One **8:** e83043

Le Bail A, Scholz S, Kost B (2013) Evaluation of reference genes for RT qPCR analyses of structure-specific and hormone regulated gene expression in Physcomitrella patens gametophytes. PLoS One **8:** e70998

Li H (2013) Aligning sequence reads, clone sequences and assembly contigs with BWA-MEM. arXiv **1303.3997**

Liao Y, Smyth GK, Shi W (2014) featureCounts: an efficient general purpose program for assigning sequence reads to genomic features. Bioinformatics **30:** 923–930

Lichtenthaler HK (1987) Chlorophylls and carotenoids: Pigments of photosynthetic biomembranes. Methods in Enzymology **148:** 350–382

Liu Y, Duan X, Zhao X, Ding W, Wang Y, Xiong Y (2021) Diverse nitrogen signals activate convergent ROP2-TOR signaling in Arabidopsis. Dev Cell **56:** 1283–1295 e1285

Livak KJ, Schmittgen TD (2001) Analysis of relative gene expression data using real-time quantitative PCR and the 2(-Delta Delta C(T)) Method. Methods **25:** 402–408

Martin M Cutadapt Removes Adapter Sequences From High-Throughput Sequencing Reads. EMBnet.Journal **17.1**

Moody LA, Kelly S, Clayton R, Weeks Z, Emms DM, Langdale JA (2021) NO GAMETOPHORES 2 Is a Novel Regulator of the 2D to 3D Growth Transition in the Moss Physcomitrella patens. Curr Biol **31:** 555–563 e554

Ortiz-Ramirez C, Hernandez-Coronado M, Thamm A, Catarino B, Wang M, Dolan L, Feijo JA, Becker JD (2016) A Transcriptome Atlas of Physcomitrella patens Provides Insights into the Evolution and Development of Land Plants. Mol Plant **9:** 205–220

Porra RJ, Thompson, W.A., Kriedemann, P.E. (1989) Determination of accurate extinction coefficients and simultaneous equations for assaying chlorophylls a and b extracted with four different solvents: verification of the concentration of chlorophyll standards by atomic absorption spectroscopy. Biochimica et Biophysica Acta (BBA) - Bioenergetics **975:** 384–394

Quinlan AR, Hall IM (2010) BEDTools: a flexible suite of utilities for comparing genomic features. Bioinformatics **26:** 841–842

Rhie A, Walenz BP, Koren S, Phillippy AM (2020) Merqury: reference-free quality, completeness, and phasing assessment for genome assemblies. Genome Biol **21:** 245

Rittenberg D, Foster L (1940) A new procedure for quantitative analysis by isotope dilution, with application to the determination of amino acids and fatty acids. J. Biol. Chem. **133:** 727–744

Robinson JT, Thorvaldsdottir H, Winckler W, Guttman M, Lander ES, Getz G, Mesirov JP (2011) Integrative genomics viewer. Nat Biotechnol **29:** 24–26

Ruffel S, Krouk G, Ristova D, Shasha D, Birnbaum KD, Coruzzi GM (2011) Nitrogen economics of root foraging: transitive closure of the nitrate-cytokinin relay and distinct systemic signaling for N supply vs. demand. Proc Natl Acad Sci U S A **108:** 18524–18529

Sakakibara H (2025) Five unaddressed questions about cytokinin biosynthesis. J Exp Bot **76:** 1941–1949

Simura J, Antoniadi I, Siroka J, Tarkowska D, Strnad M, Ljung K, Novak O (2018) Plant Hormonomics: Multiple Phytohormone Profiling by Targeted Metabolomics. Plant Physiol **177:** 476–489

Van der Auwera GABDOC (2017) Scaling accurate genetic variant discovery to tens of thousands of samples. biorxv **201178**

Winter D, Vinegar B, Nahal H, Ammar R, Wilson GV, Provart NJ (2007) An "Electronic Fluorescent Pictograph" browser for exploring and analyzing large-scale biological data sets. PLoS One **2:** e718

Wu T, Hu E, Xu S, Chen M, Guo P, Dai Z, Feng T, Zhou L, Tang W, Zhan L, Fu X, Liu S, Bo X, Yu G (2021) clusterProfiler 4.0: A universal enrichment tool for interpreting omics data. Innovation (Camb) **2:** 100141
